# Supplementary figures and images for: Age grading An. gambiae and An. arabiensis using near infrared spectra and artificial neural networks
Source: PLoS One. 2019 Aug 14;14(8):e0209451. doi: 10.1371/journal.pone.0209451 (PMC6693756; doi:10.1371/journal.pone.0209451)

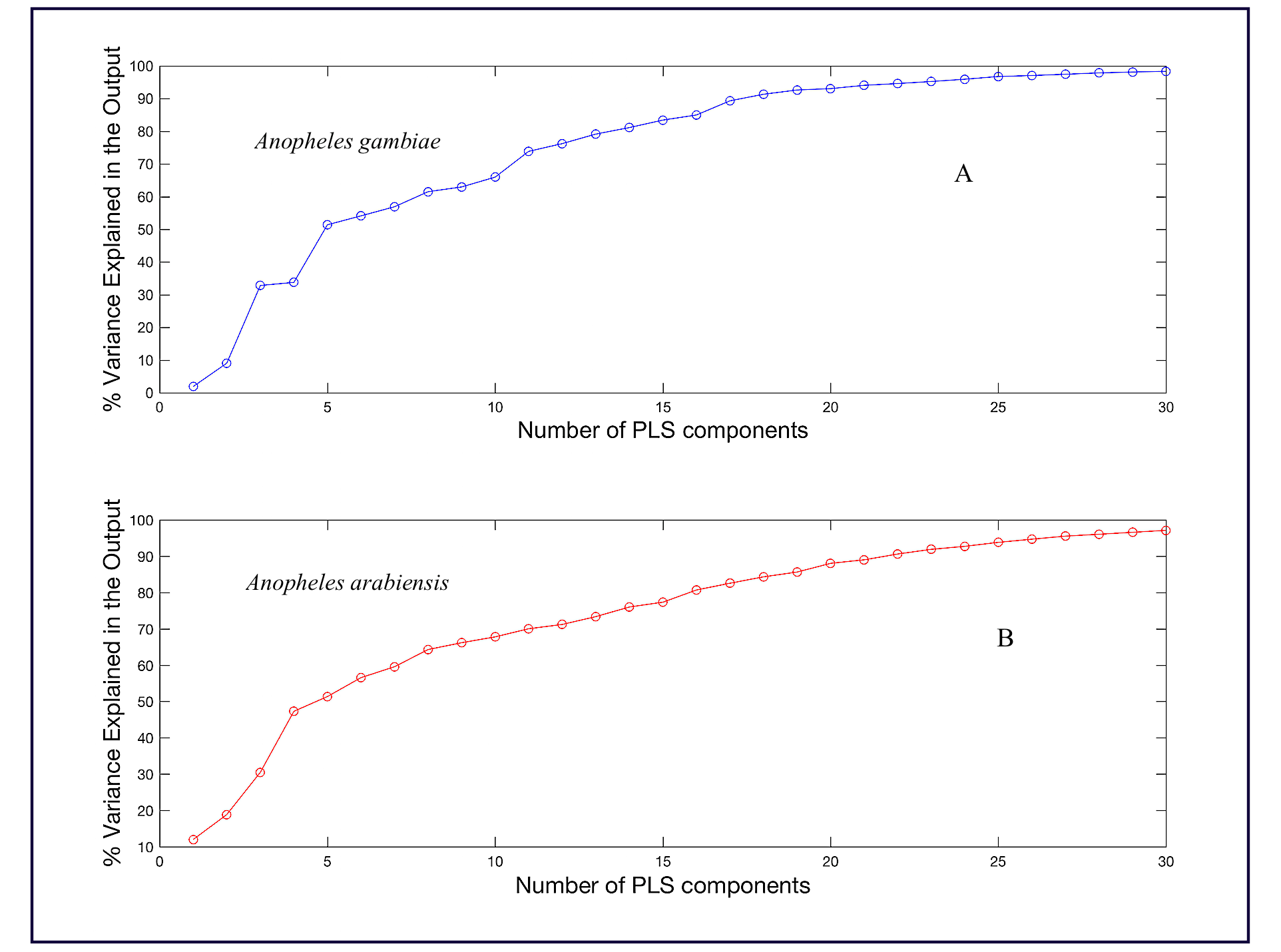

Supplement: S1 Fig — (TIF) [file pone.0209451.s001.tif]

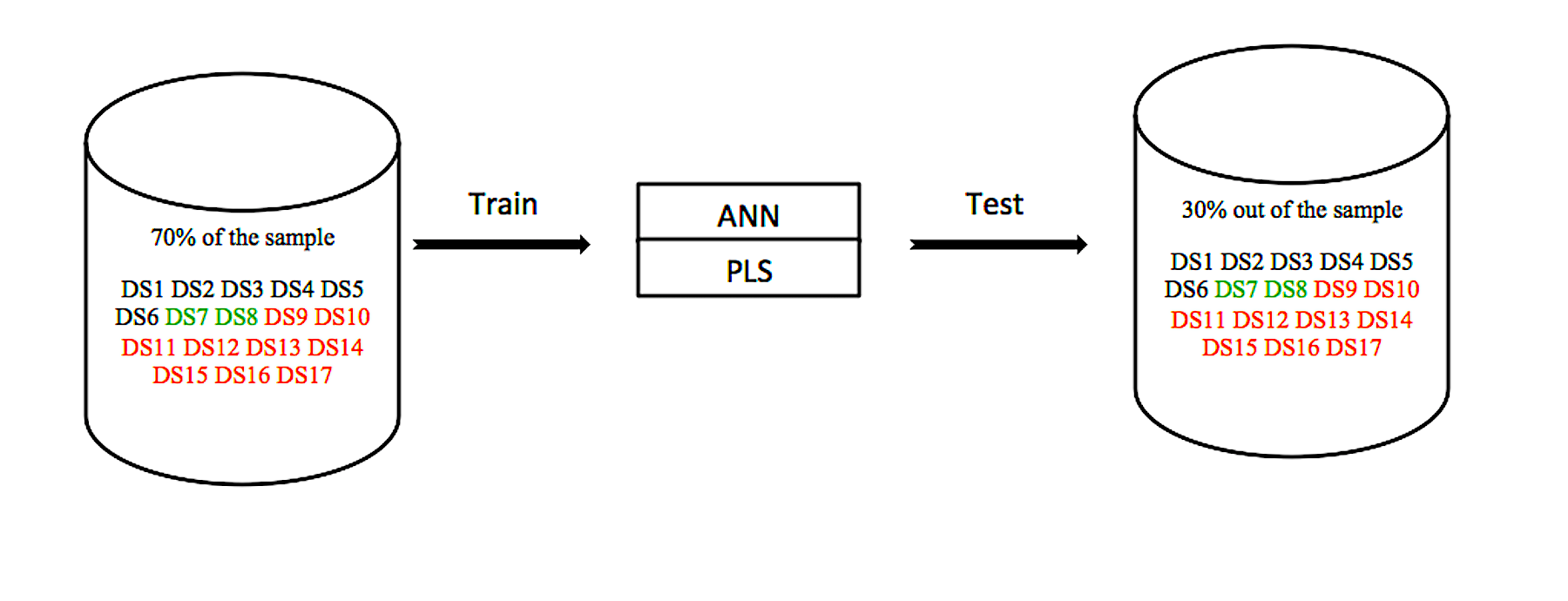

Supplement: S2 Fig — (TIFF) [file pone.0209451.s002.tiff]

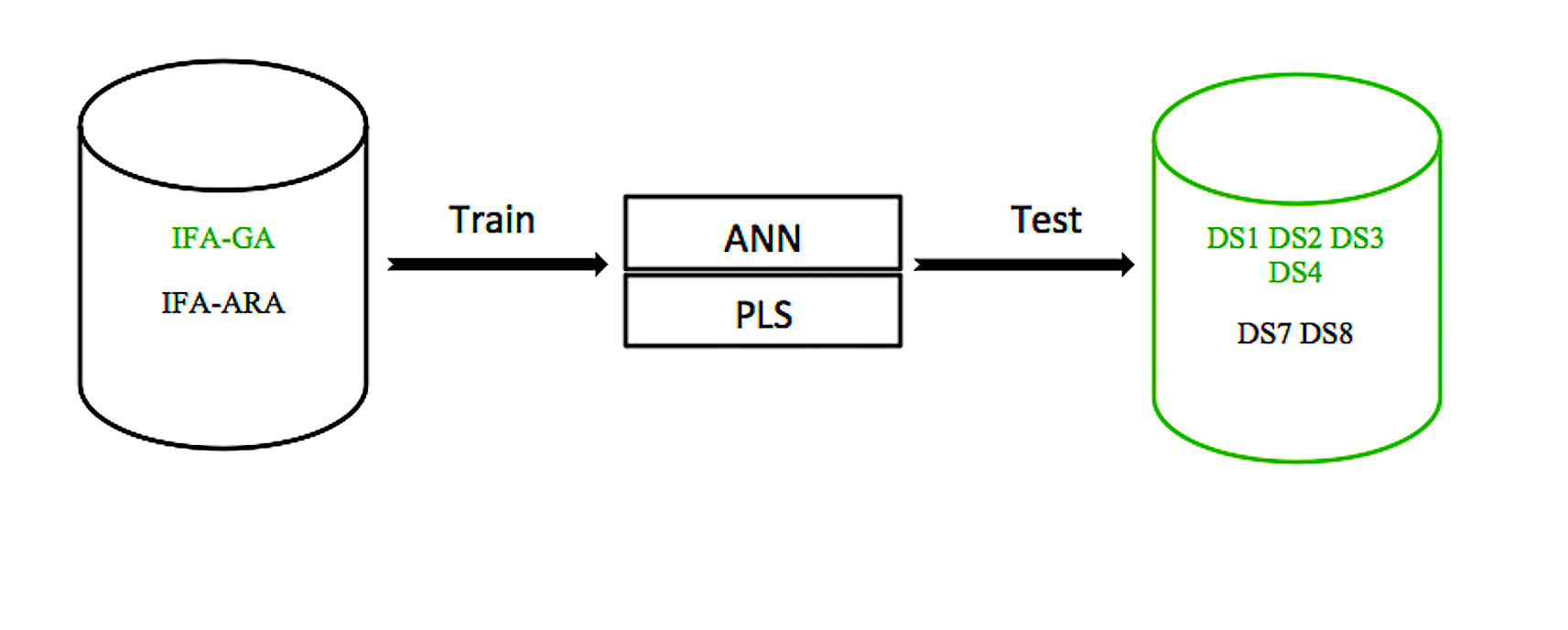

Supplement: S3 Fig — (TIFF) [file pone.0209451.s003.tiff]

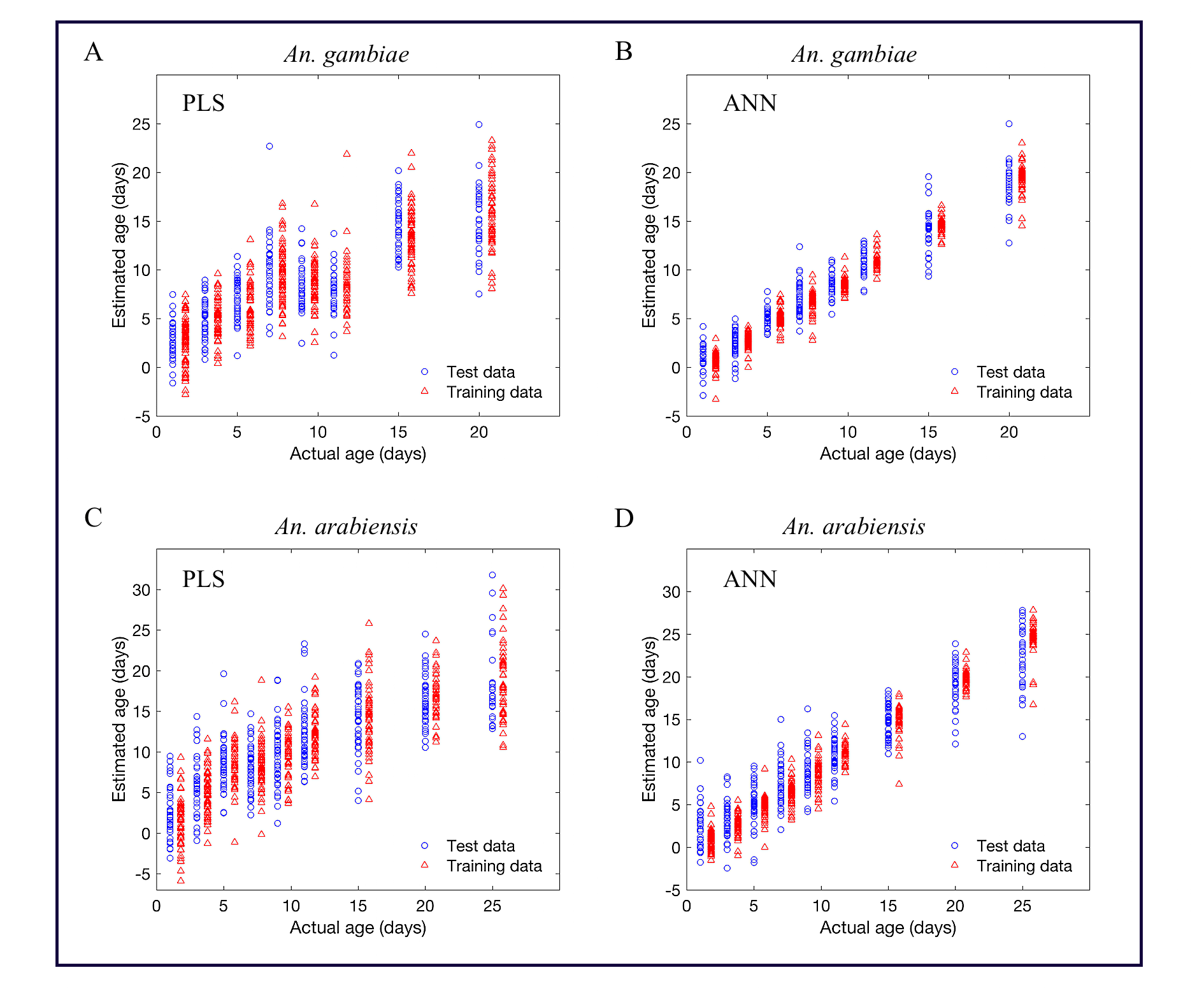

Supplement: S4 Fig — (TIF) [file pone.0209451.s004.tif]

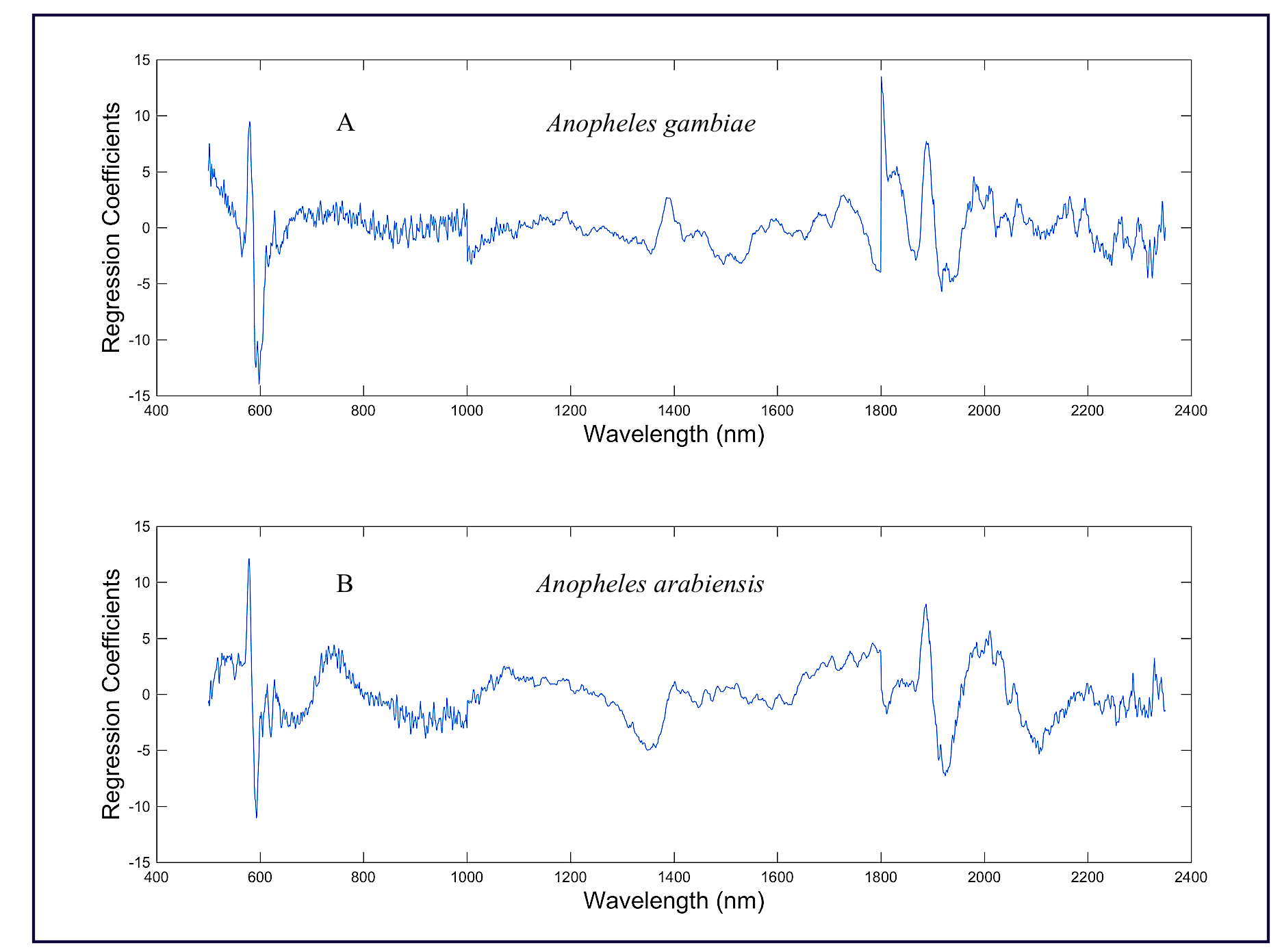

Supplement: S5 Fig — (TIF) [file pone.0209451.s005.tif]

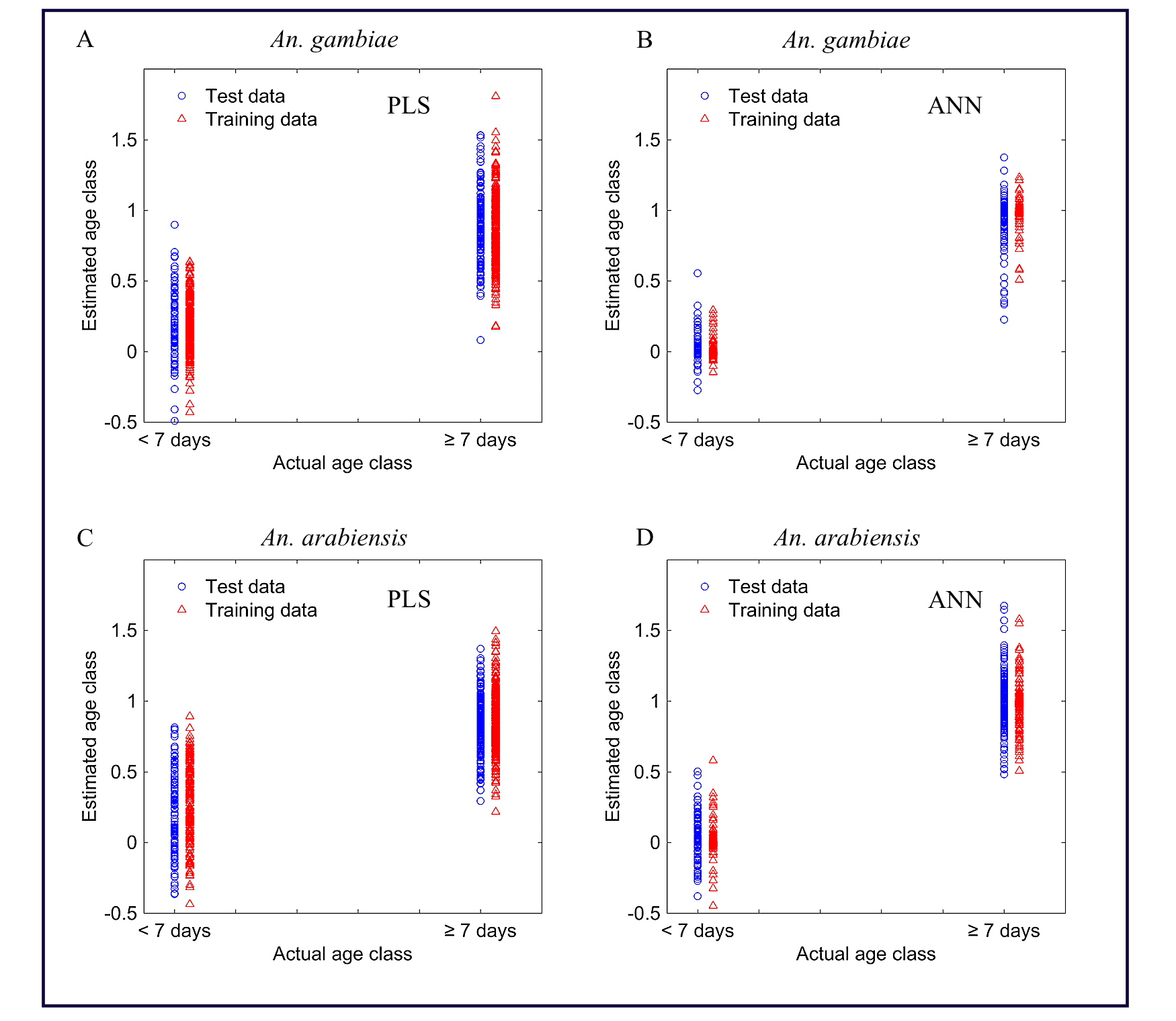

Supplement: S6 Fig — (TIF) [file pone.0209451.s006.tif]

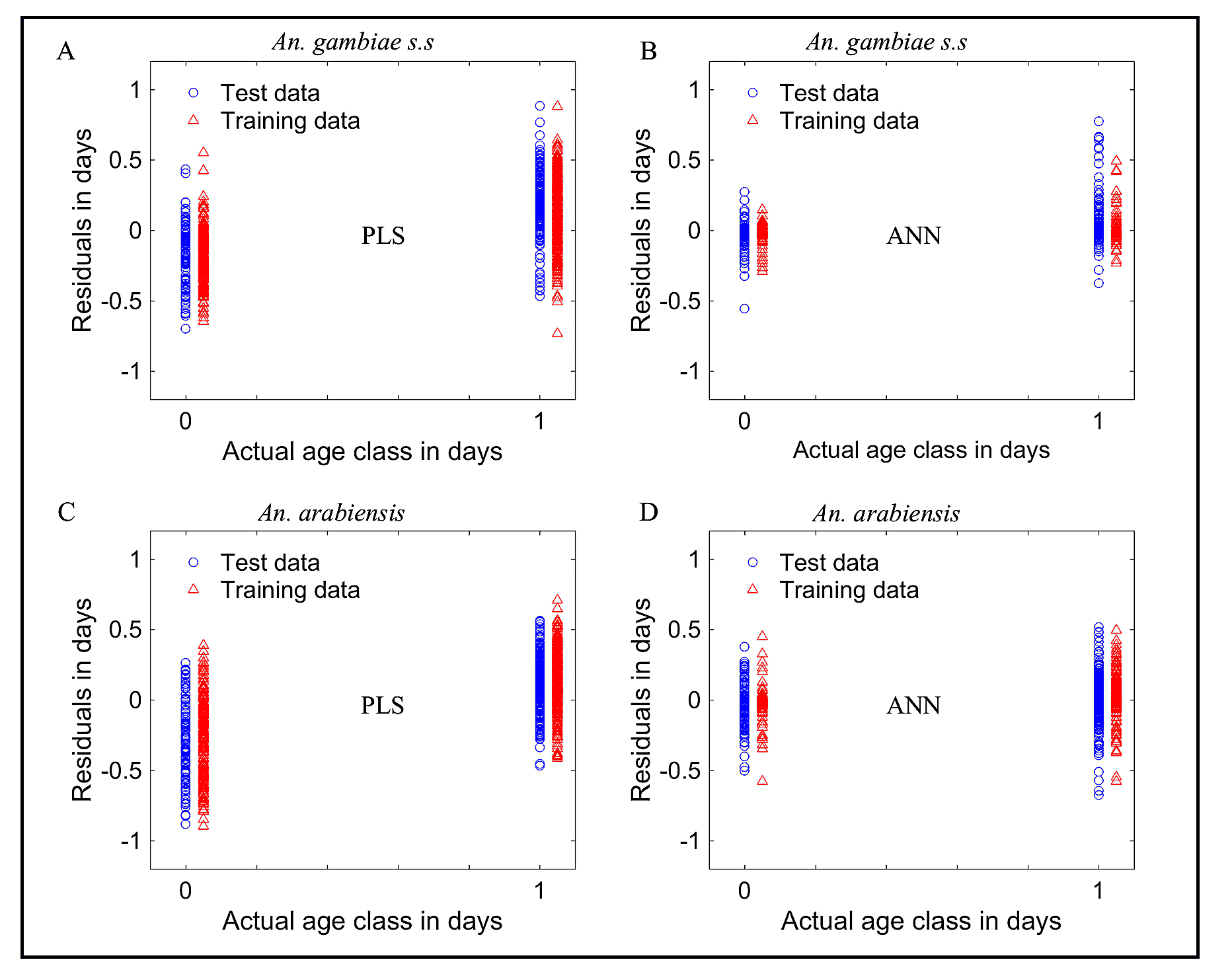

Supplement: S7 Fig — (TIF) [file pone.0209451.s007.tif]

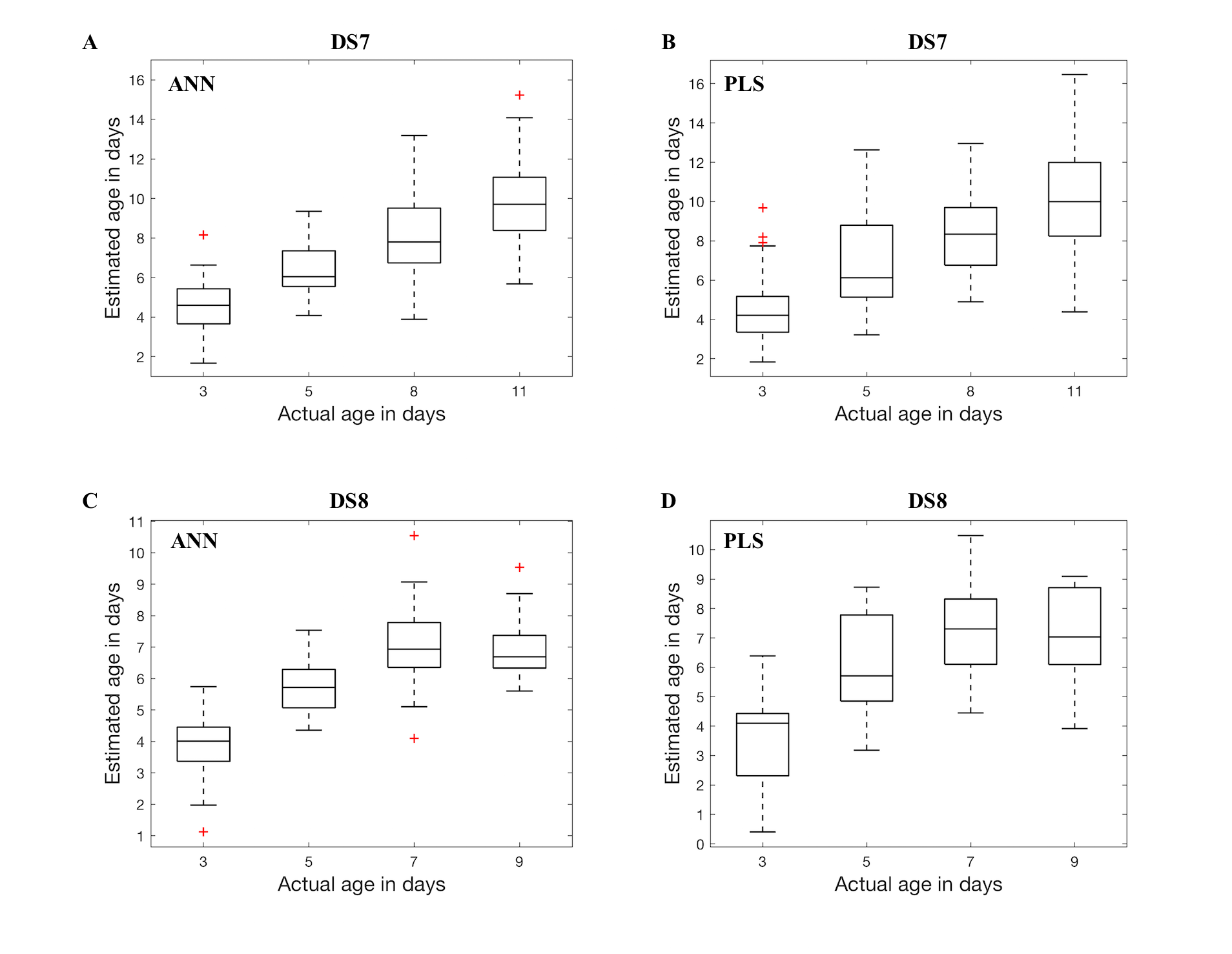

Supplement: S6 Appendix — (ZIP) [file pone.0209451.s022.zip › S6_Appendix/S10_Fig_DS7_To_DS8.tif]

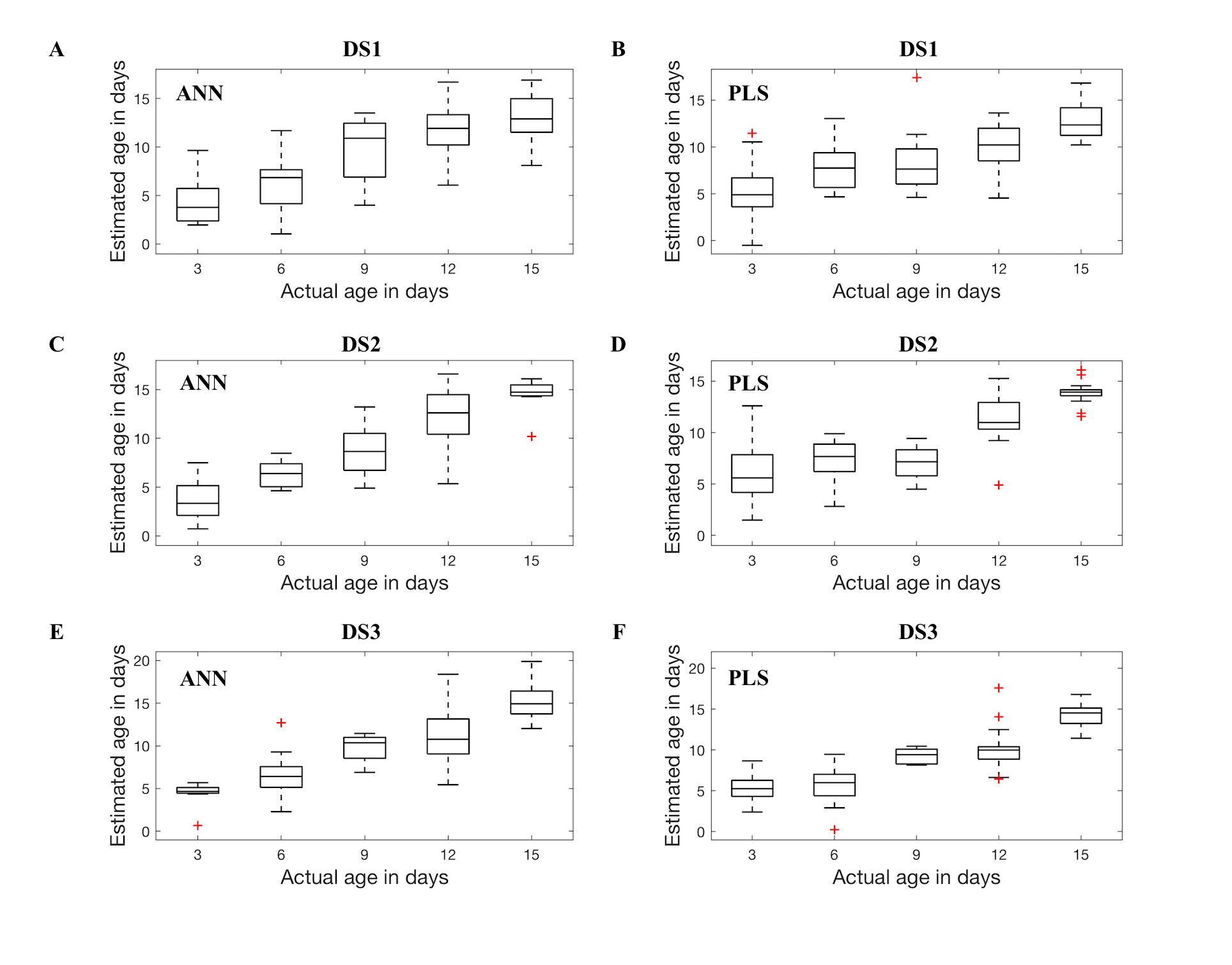

Supplement: S6 Appendix — (ZIP) [file pone.0209451.s022.zip › S6_Appendix/S8_Fig_DS1_To_DS3.tif]

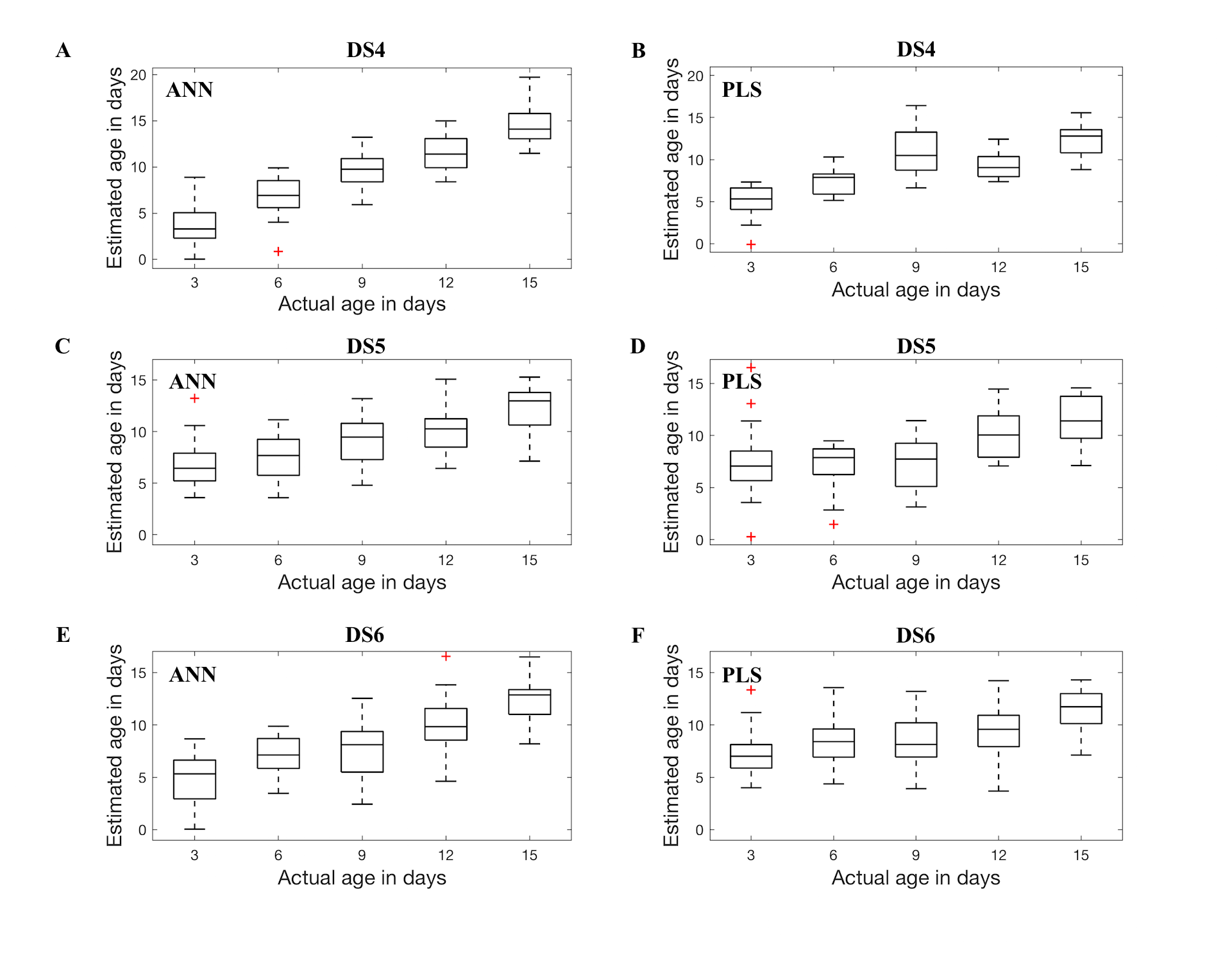

Supplement: S6 Appendix — (ZIP) [file pone.0209451.s022.zip › S6_Appendix/S9_Fig_DS4_To_DS6.tif]

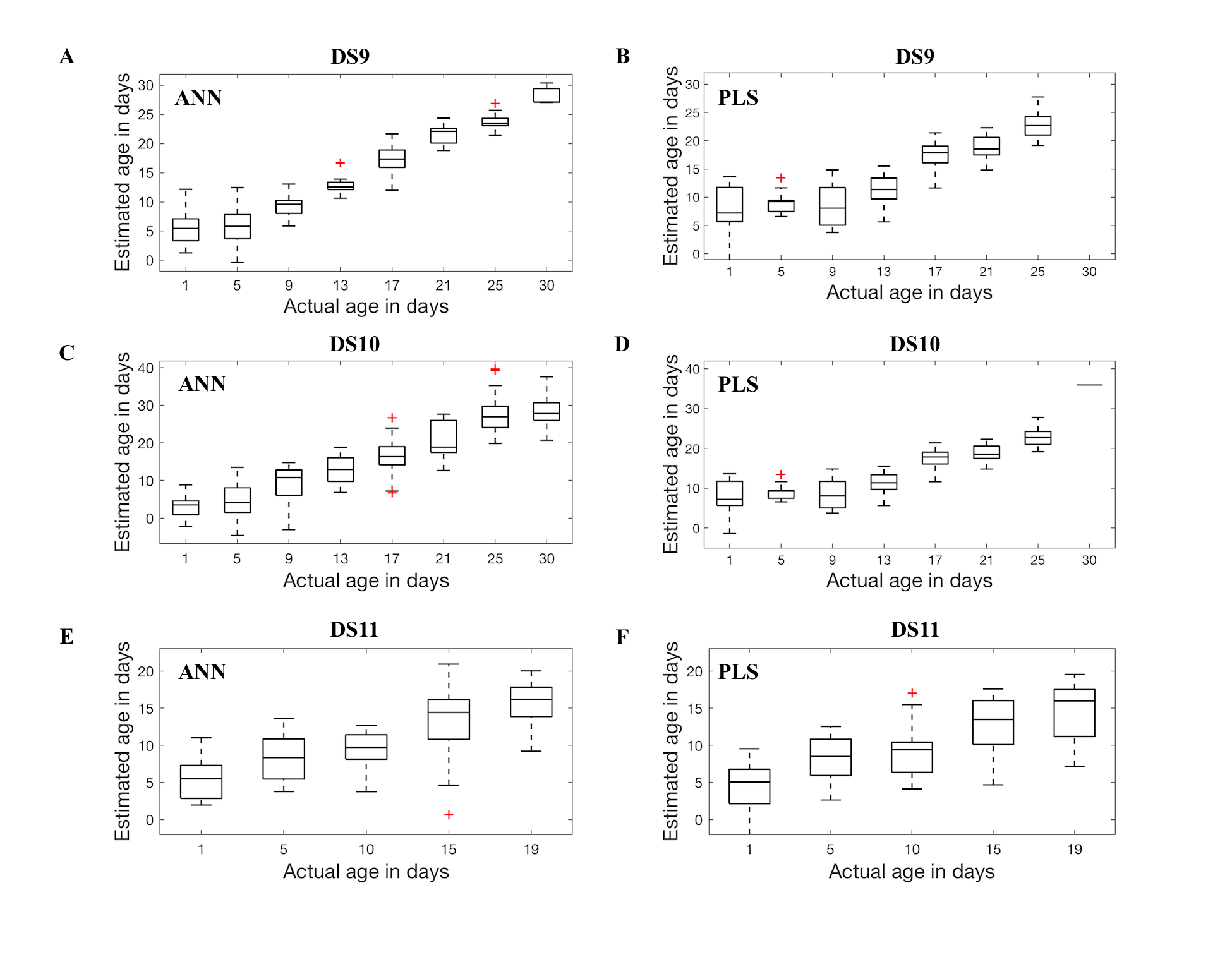

Supplement: S7 Appendix — (ZIP) [file pone.0209451.s023.zip › S7_Appendix/S11_Fig_DS9_To_DS11.tif]

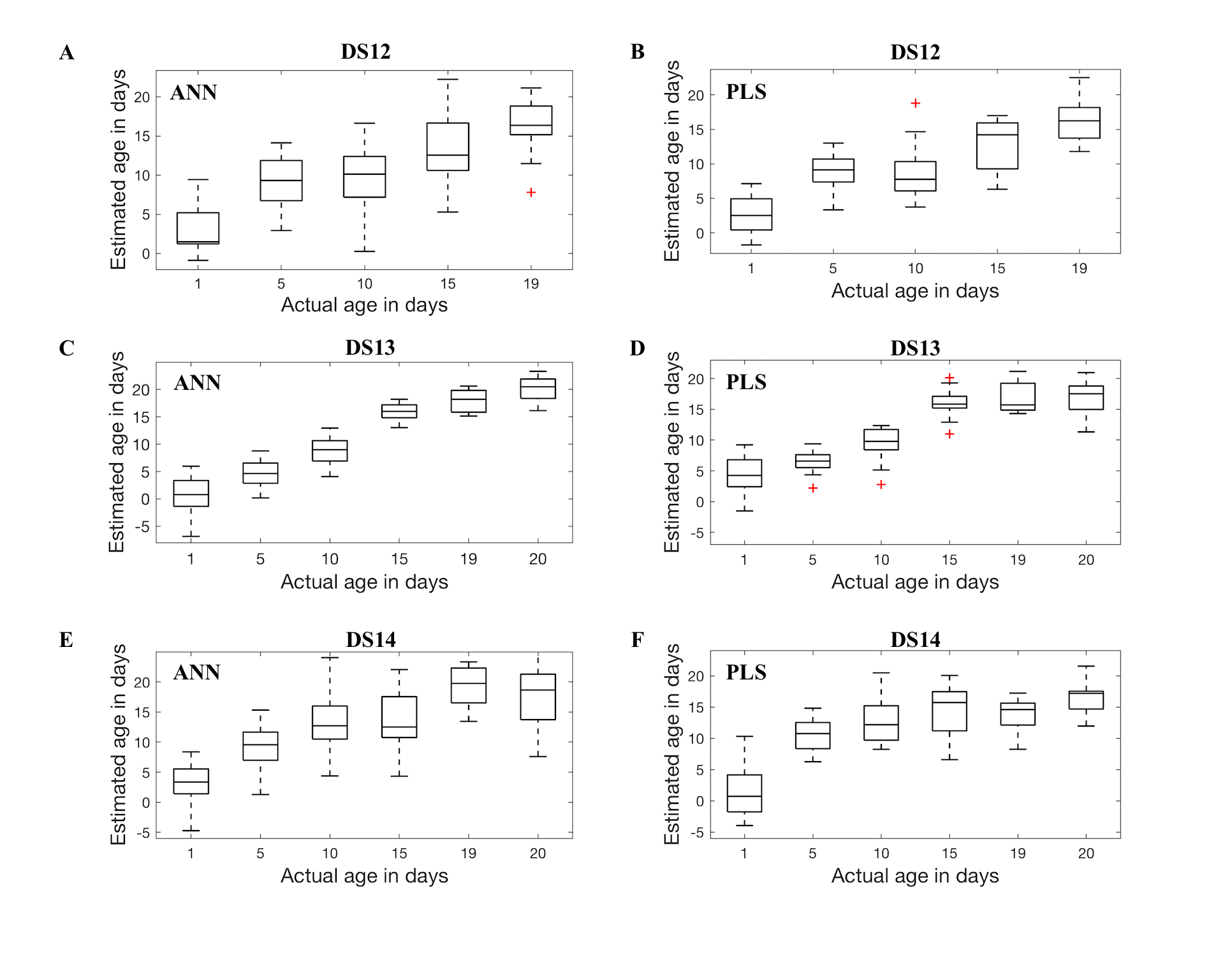

Supplement: S7 Appendix — (ZIP) [file pone.0209451.s023.zip › S7_Appendix/S12_Fig_DS12_To_DS14.tif]

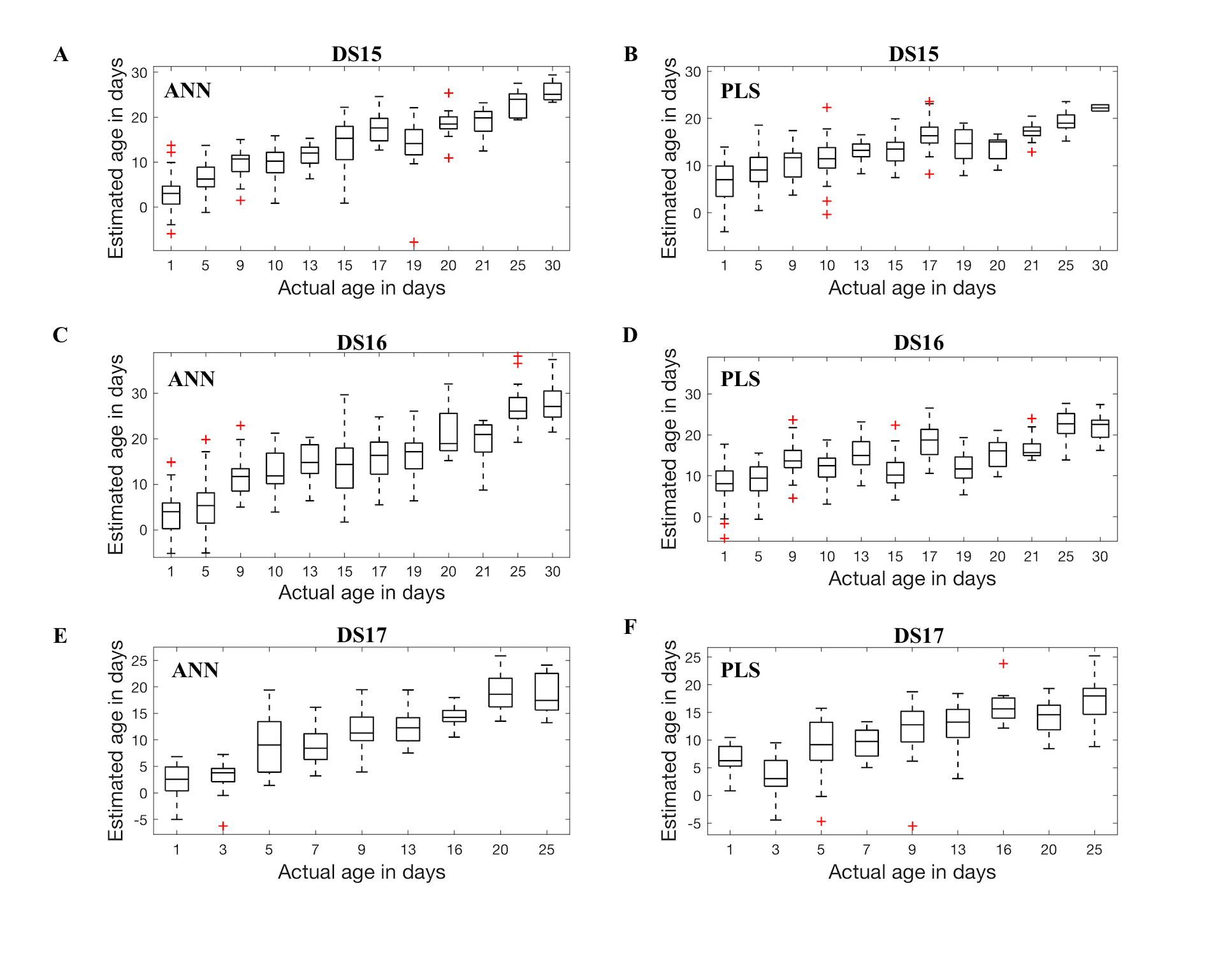

Supplement: S7 Appendix — (ZIP) [file pone.0209451.s023.zip › S7_Appendix/S13_Fig_DS15_To_DS17.tif]

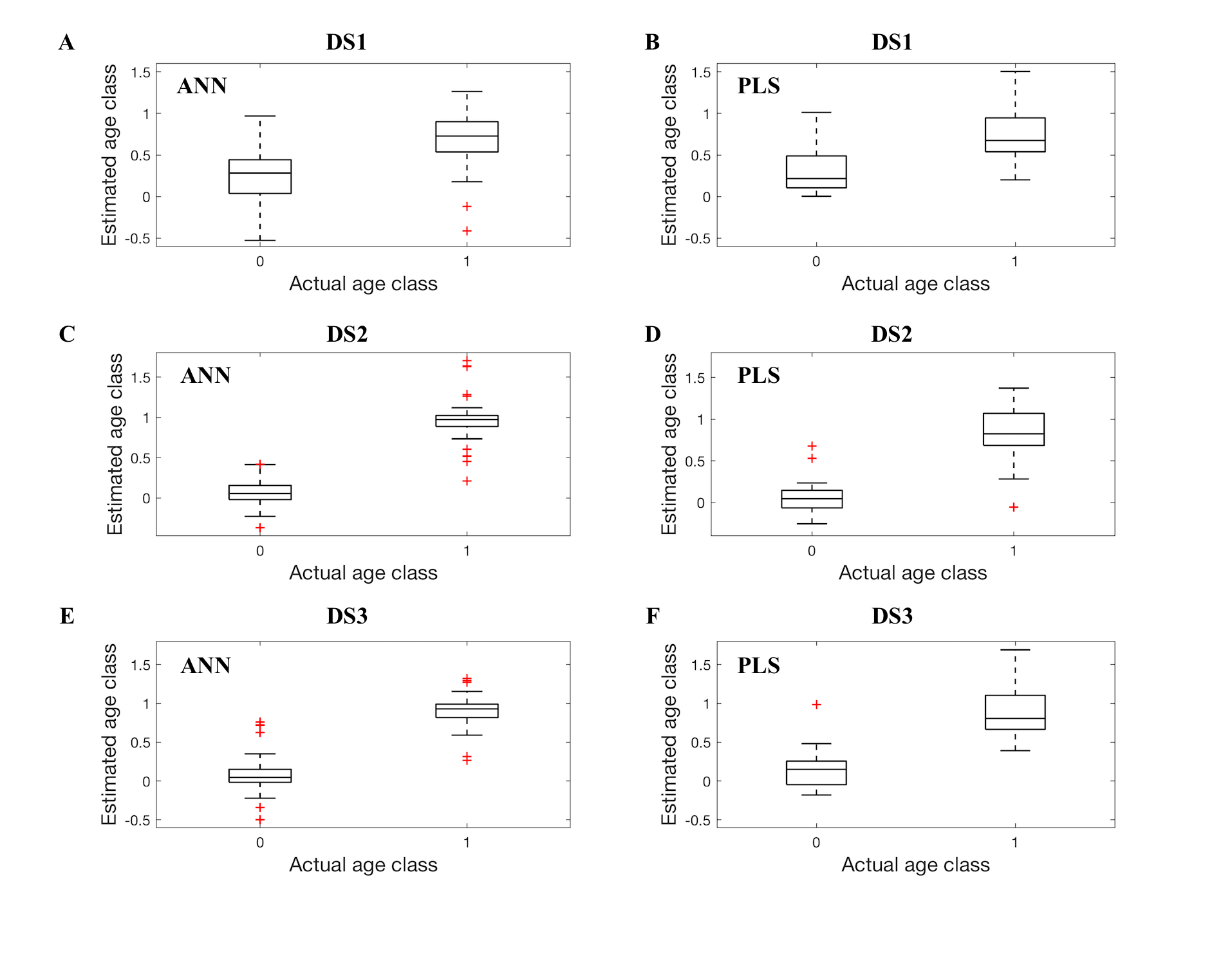

Supplement: S8 Appendix — (ZIP) [file pone.0209451.s024.zip › S8_Appendix/S14_Fig_DS1_To_DS3_Binary.tif]

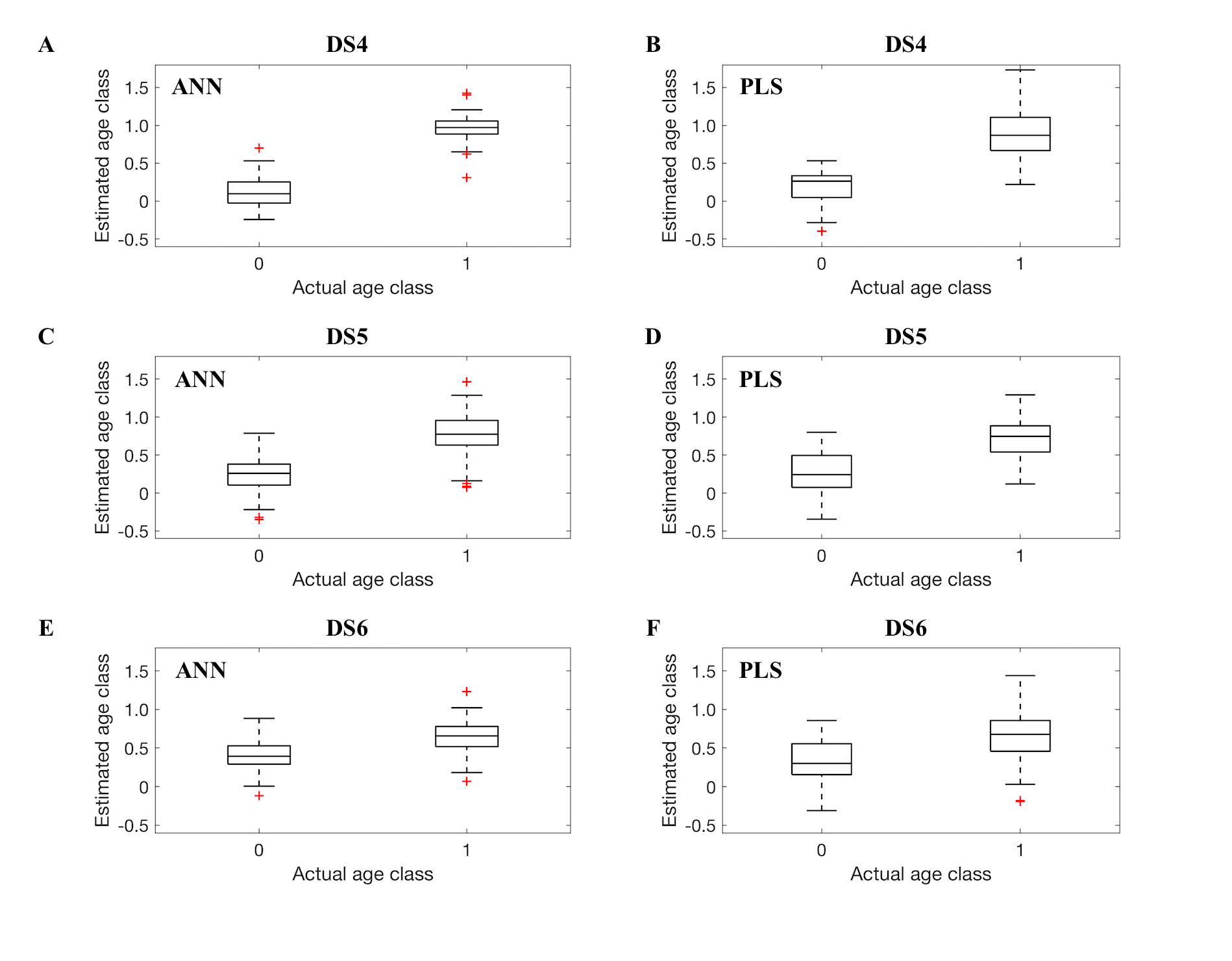

Supplement: S8 Appendix — (ZIP) [file pone.0209451.s024.zip › S8_Appendix/S15_Fig_DS4_To_DS6_Binary.tif]

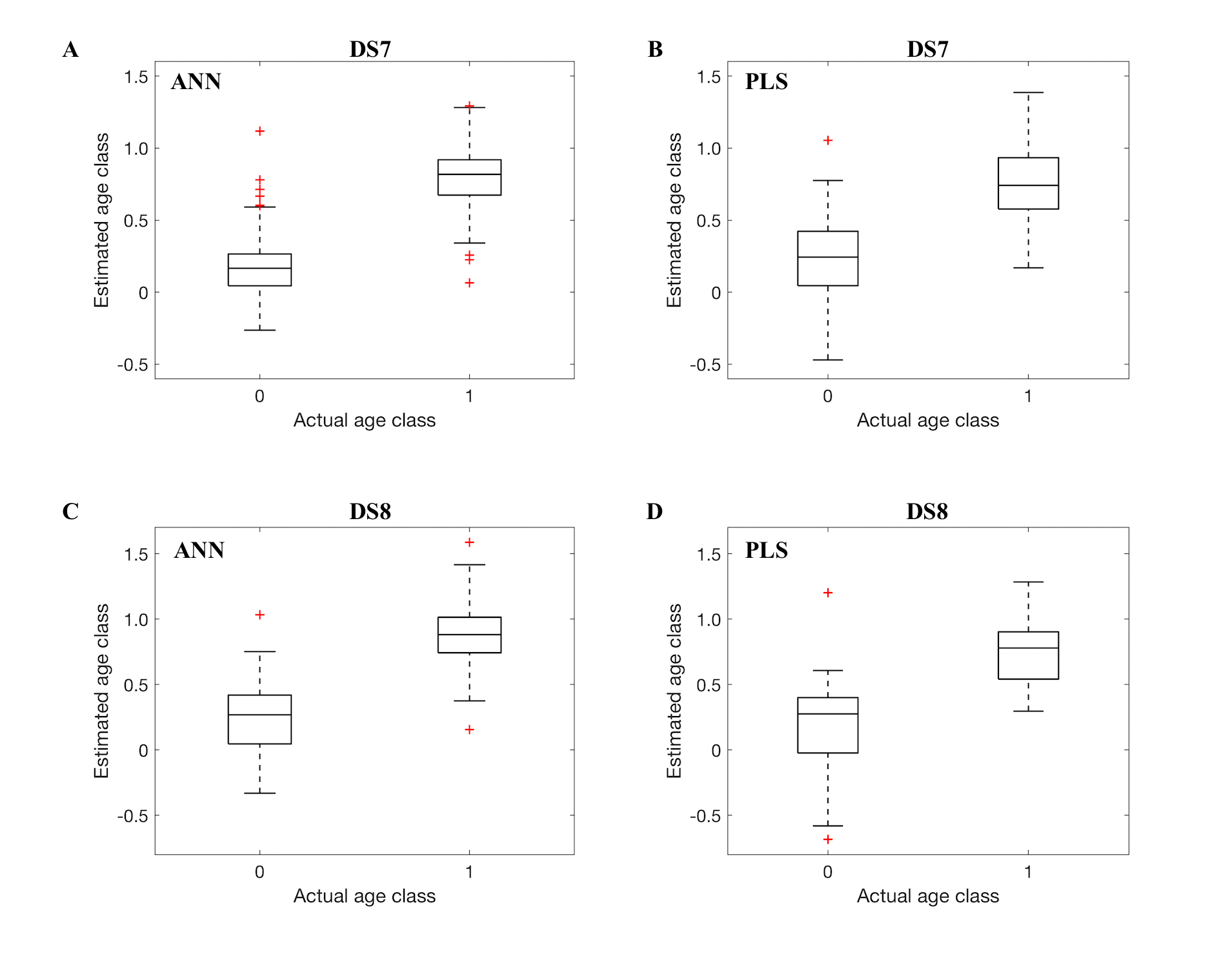

Supplement: S8 Appendix — (ZIP) [file pone.0209451.s024.zip › S8_Appendix/S16_Fig_DS7_To_DS8_Binary.tif]

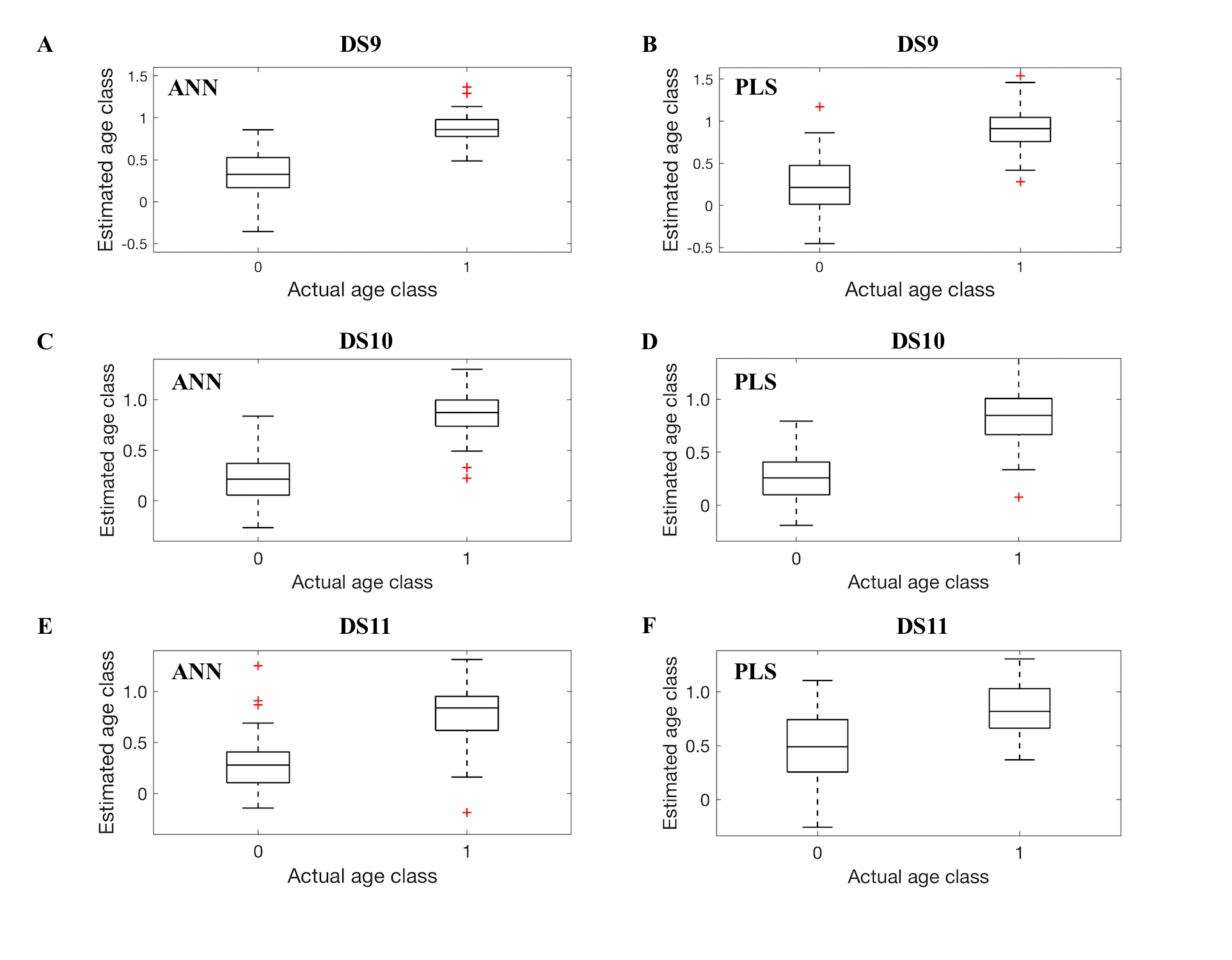

Supplement: S9 Appendix — (ZIP) [file pone.0209451.s025.zip › S9_Appendix/S17_Fig_DS9_To_DS11_Binary.tif]

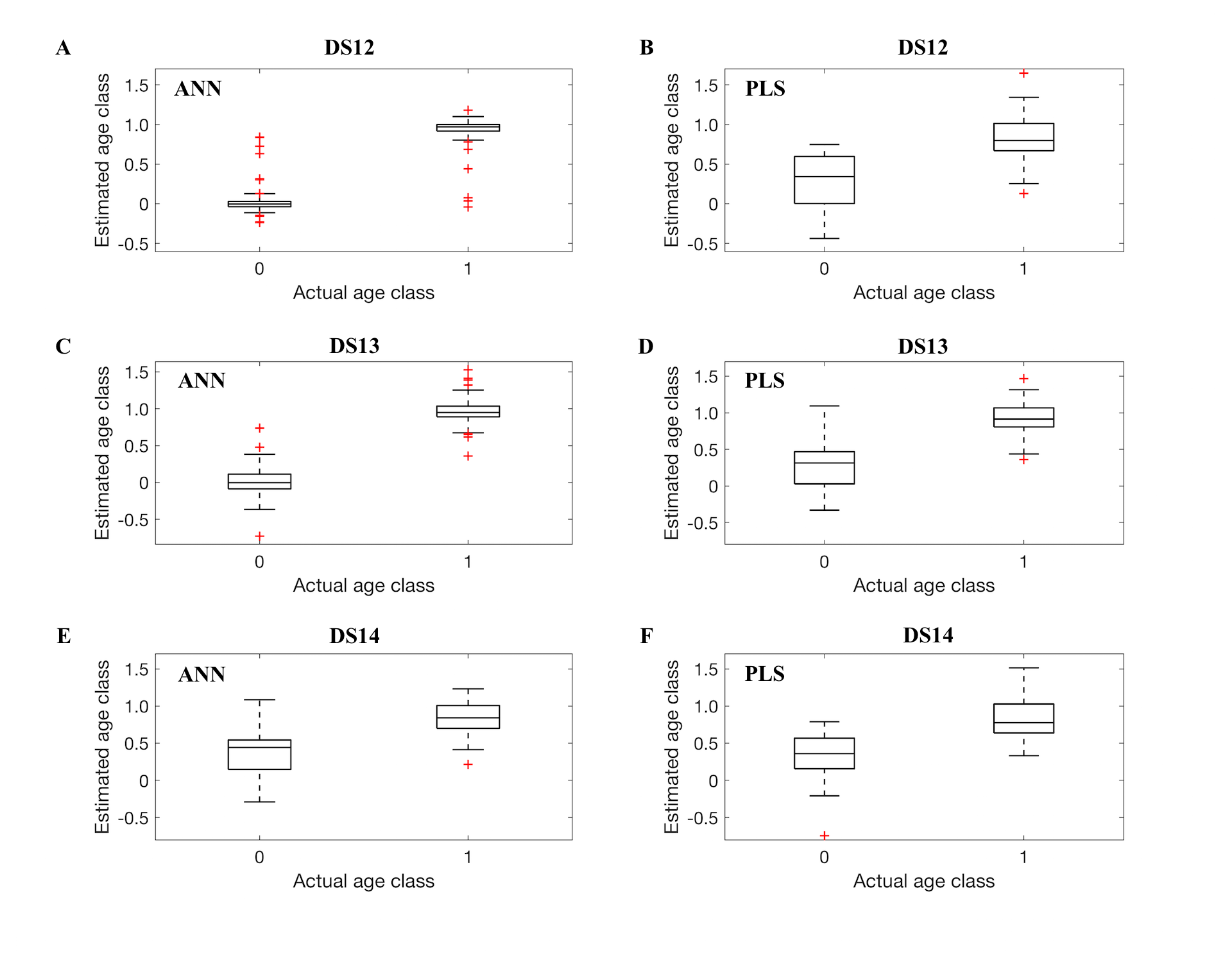

Supplement: S9 Appendix — (ZIP) [file pone.0209451.s025.zip › S9_Appendix/S18_Fig_DS12_To_DS14_Binary.tif]

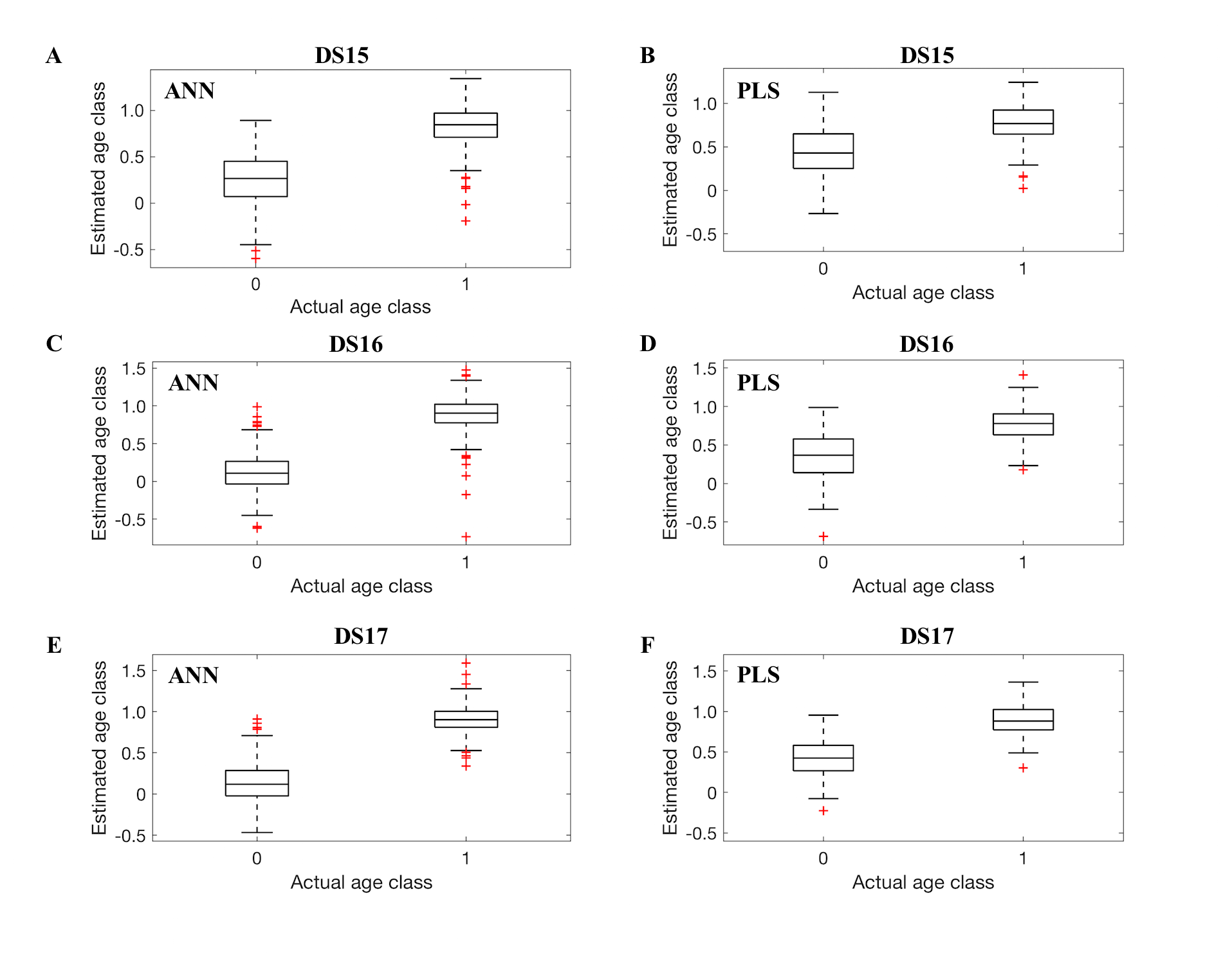

Supplement: S9 Appendix — (ZIP) [file pone.0209451.s025.zip › S9_Appendix/S19_Fig_DS15_To_DS17_Binary.tif]

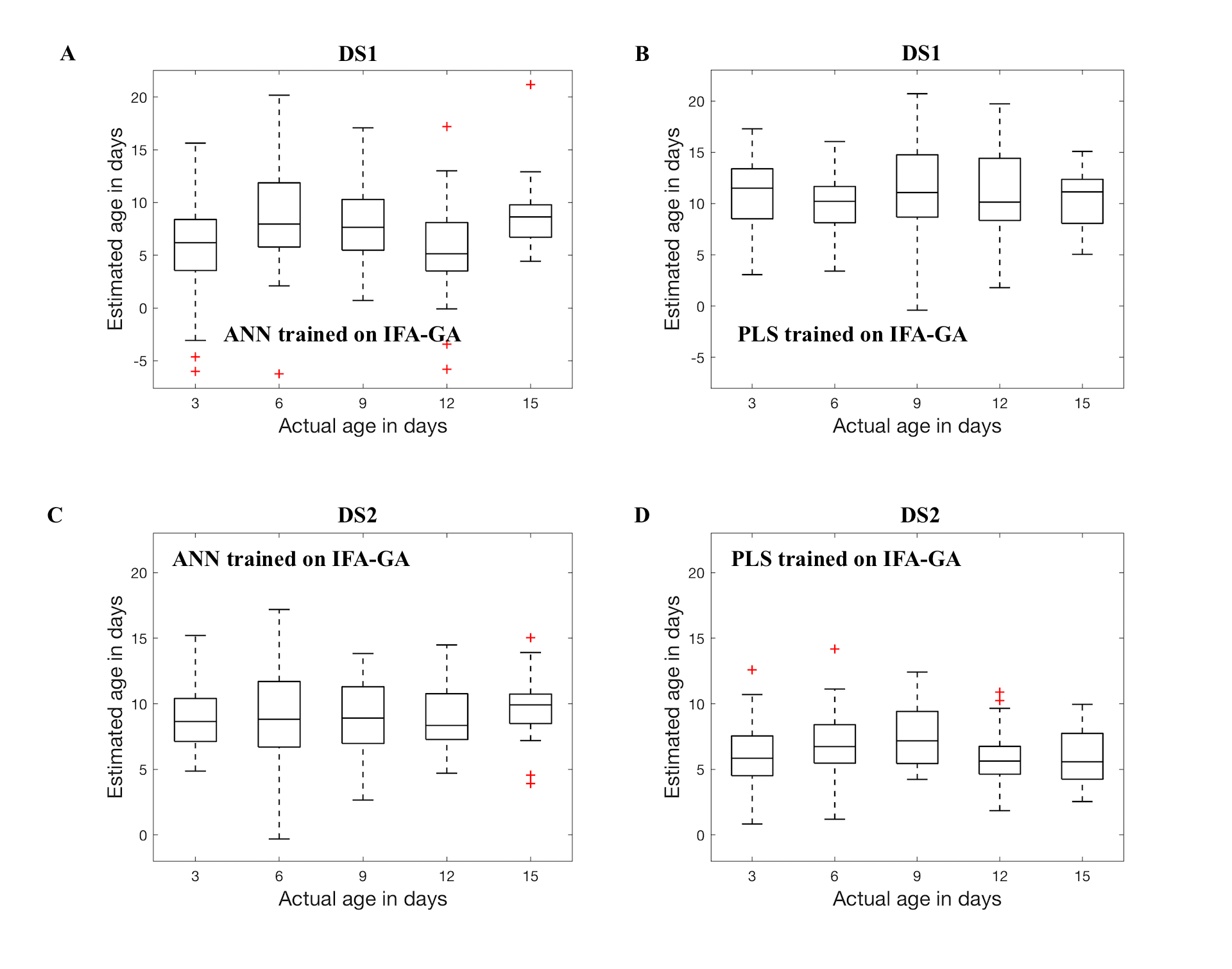

Supplement: S10 Appendix — (ZIP) [file pone.0209451.s026.zip › S10_Appendix/S20_Fig_IFA_GA_ON_DS1_AND_DS2_Regresser.tif]

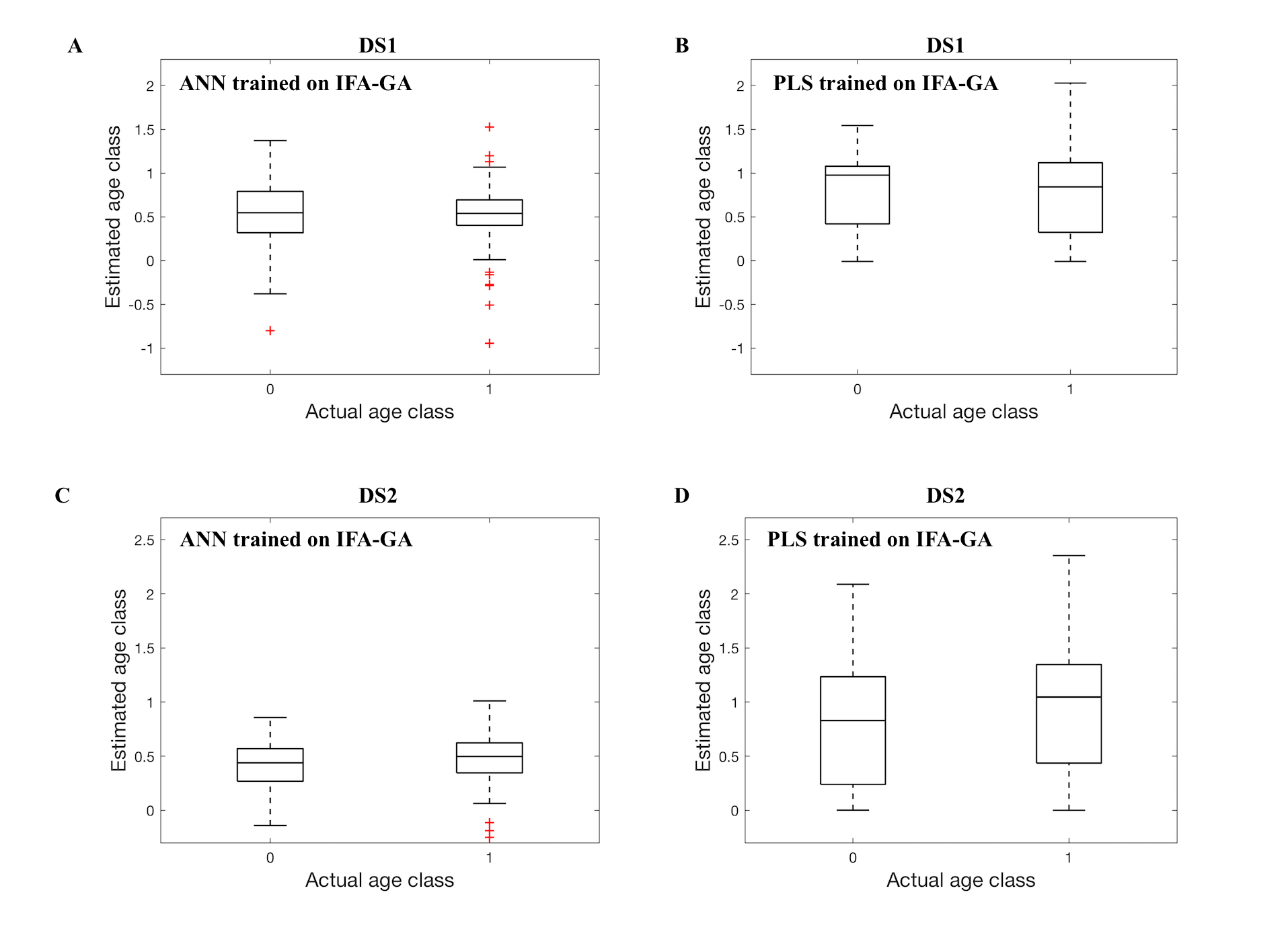

Supplement: S10 Appendix — (ZIP) [file pone.0209451.s026.zip › S10_Appendix/S21_Fig_IFA_GA_ON_DS1_AND_DS2_Binary.tif]

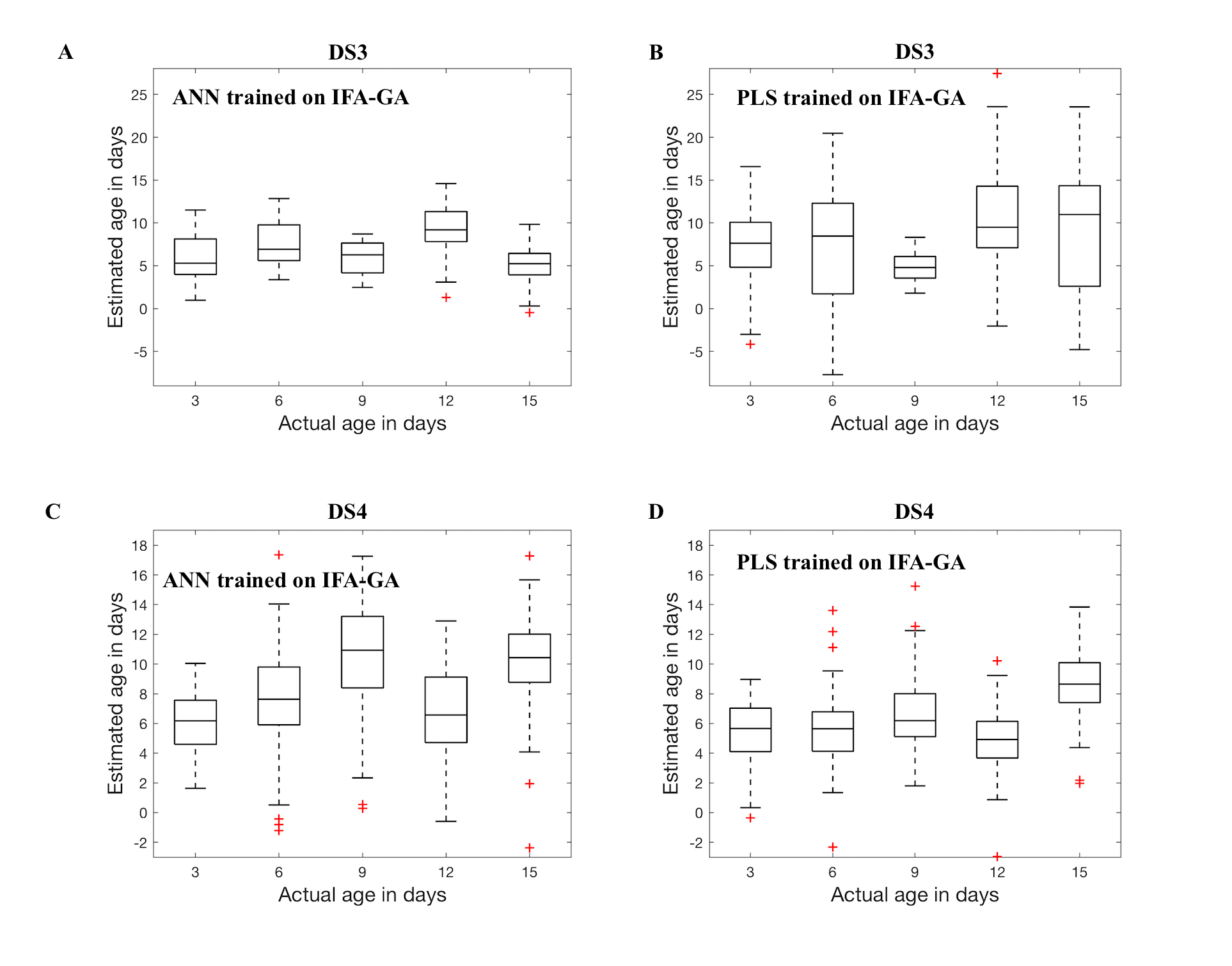

Supplement: S10 Appendix — (ZIP) [file pone.0209451.s026.zip › S10_Appendix/S22_Fig_IFA_GA_ON_DS3_AND_DS4_Regresser.tif]

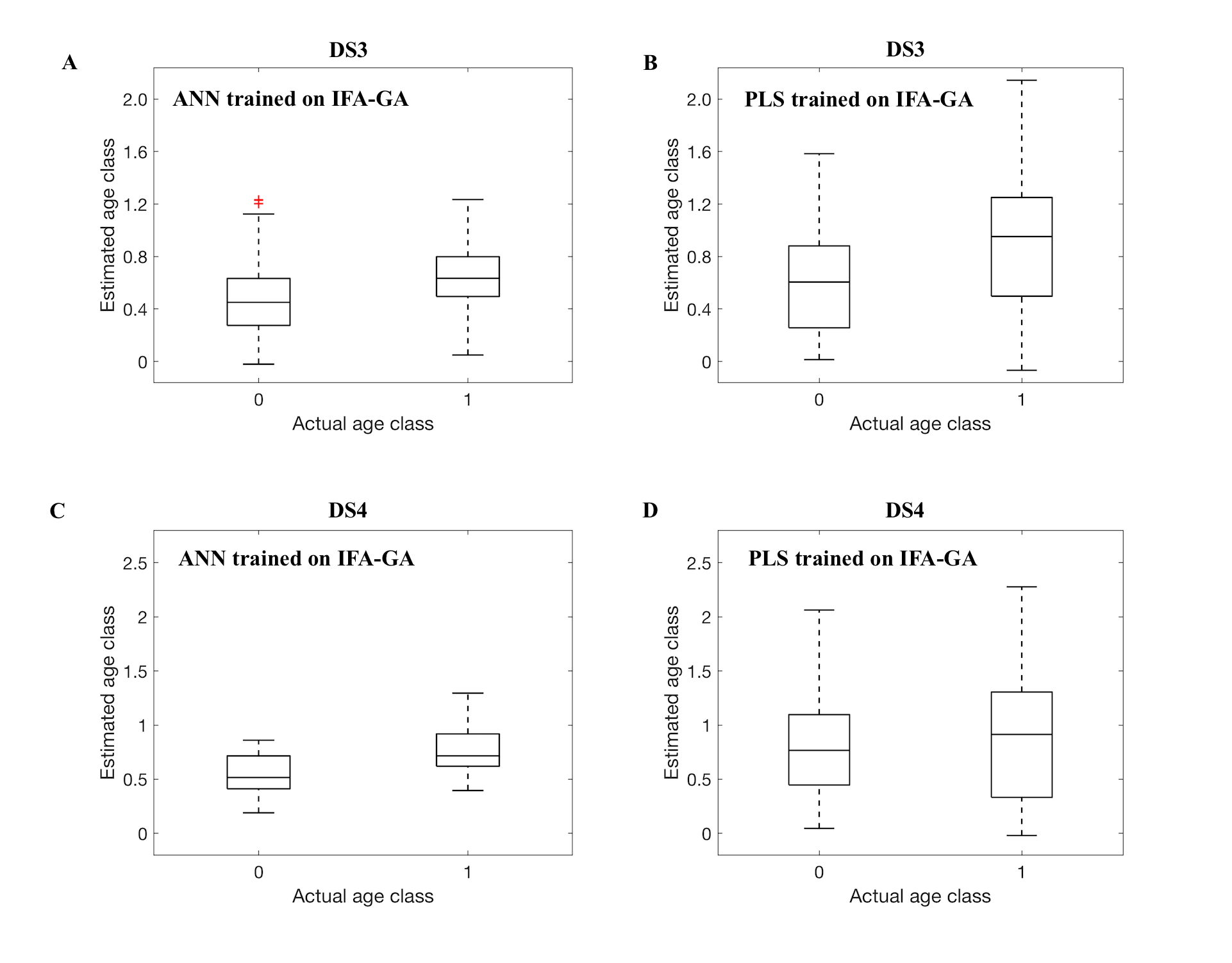

Supplement: S10 Appendix — (ZIP) [file pone.0209451.s026.zip › S10_Appendix/S23_Fig_IFA_GA_ON_DS3_AND_DS4_Binary.tif]

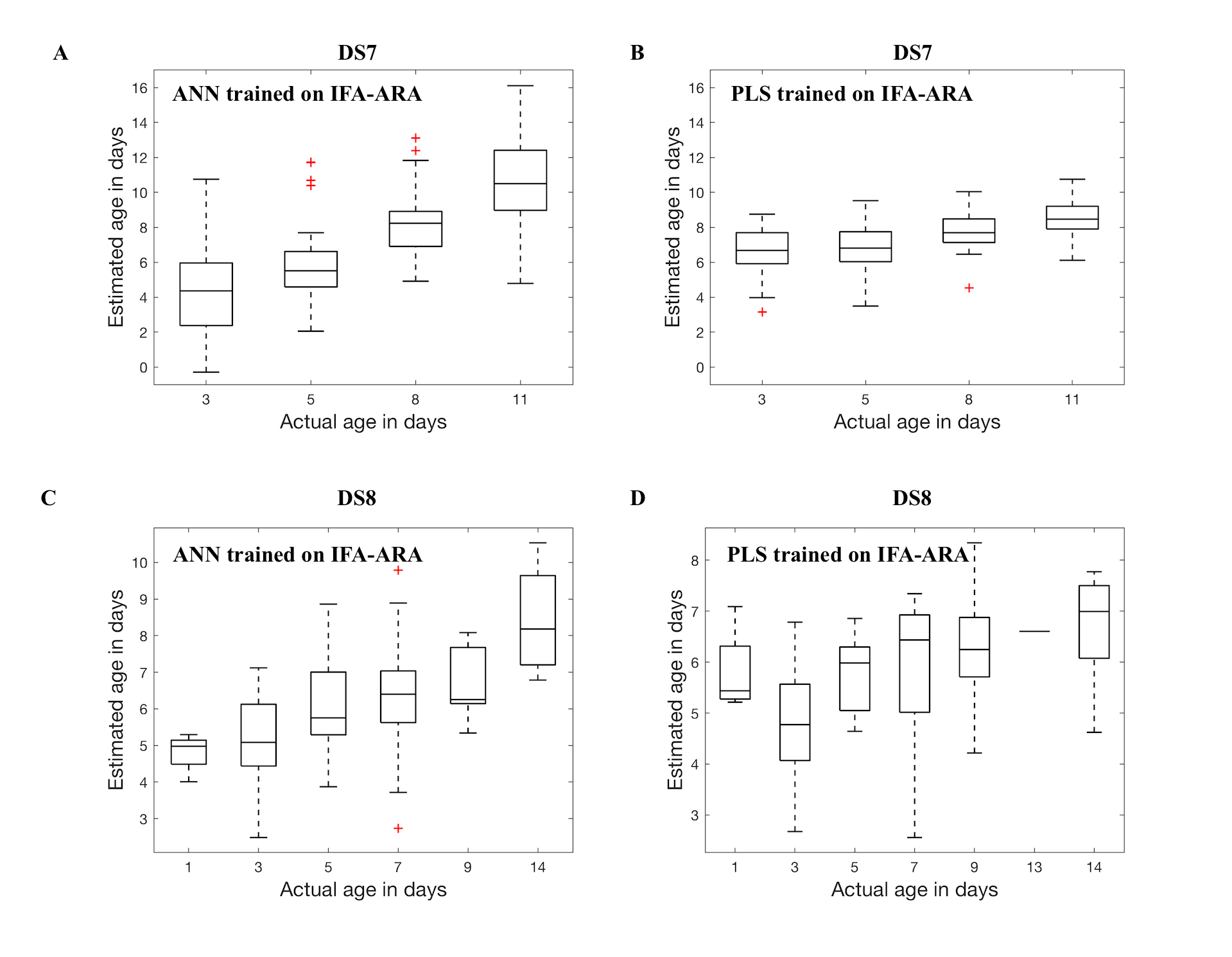

Supplement: S10 Appendix — (ZIP) [file pone.0209451.s026.zip › S10_Appendix/S24_Fig_IFA_ARA_ON_DS7_AND_DS8_Regresser.tif]

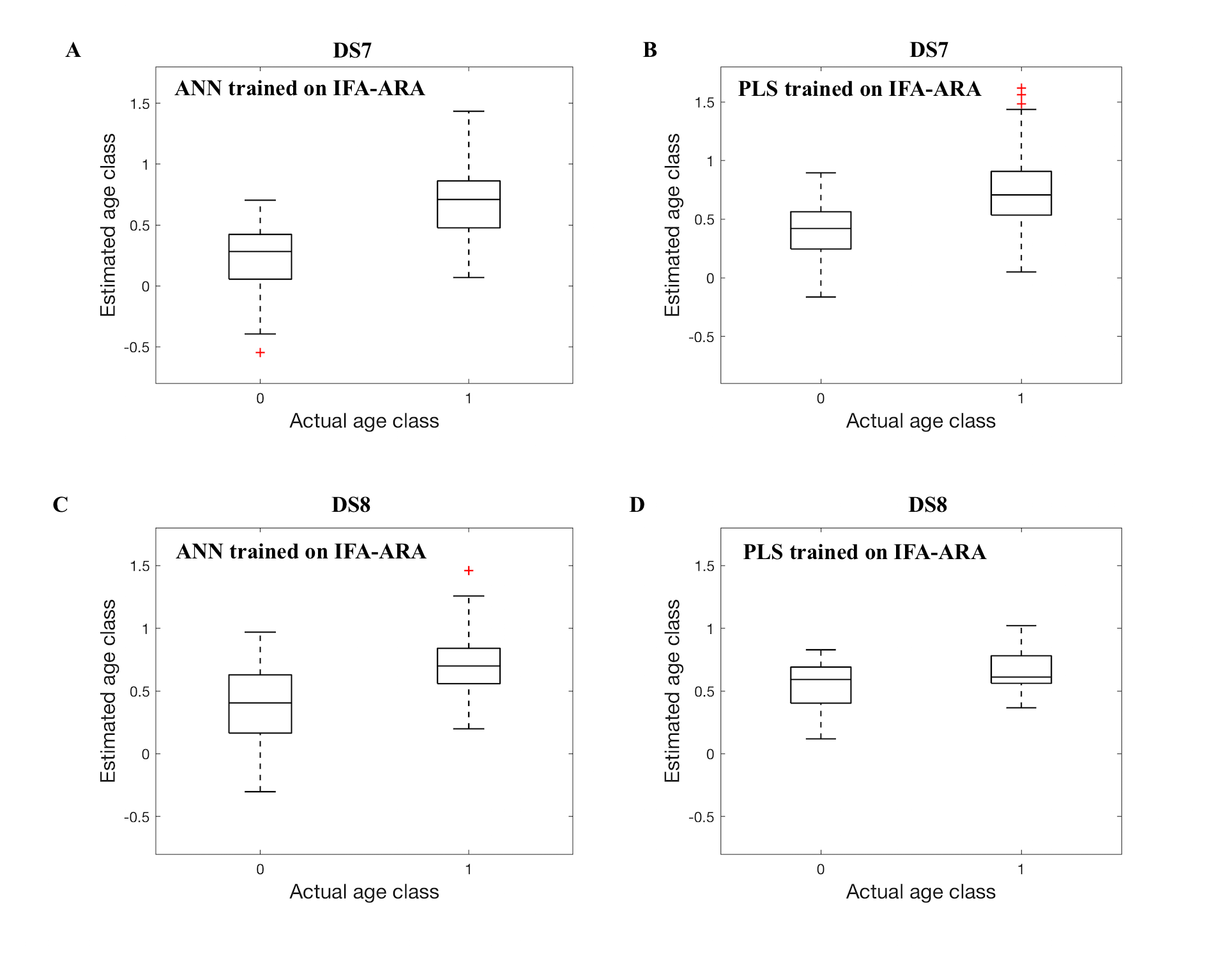

Supplement: S10 Appendix — (ZIP) [file pone.0209451.s026.zip › S10_Appendix/S25_Fig_IFA_ARA_ON_DS7_AND_DS8_Binary.tif]

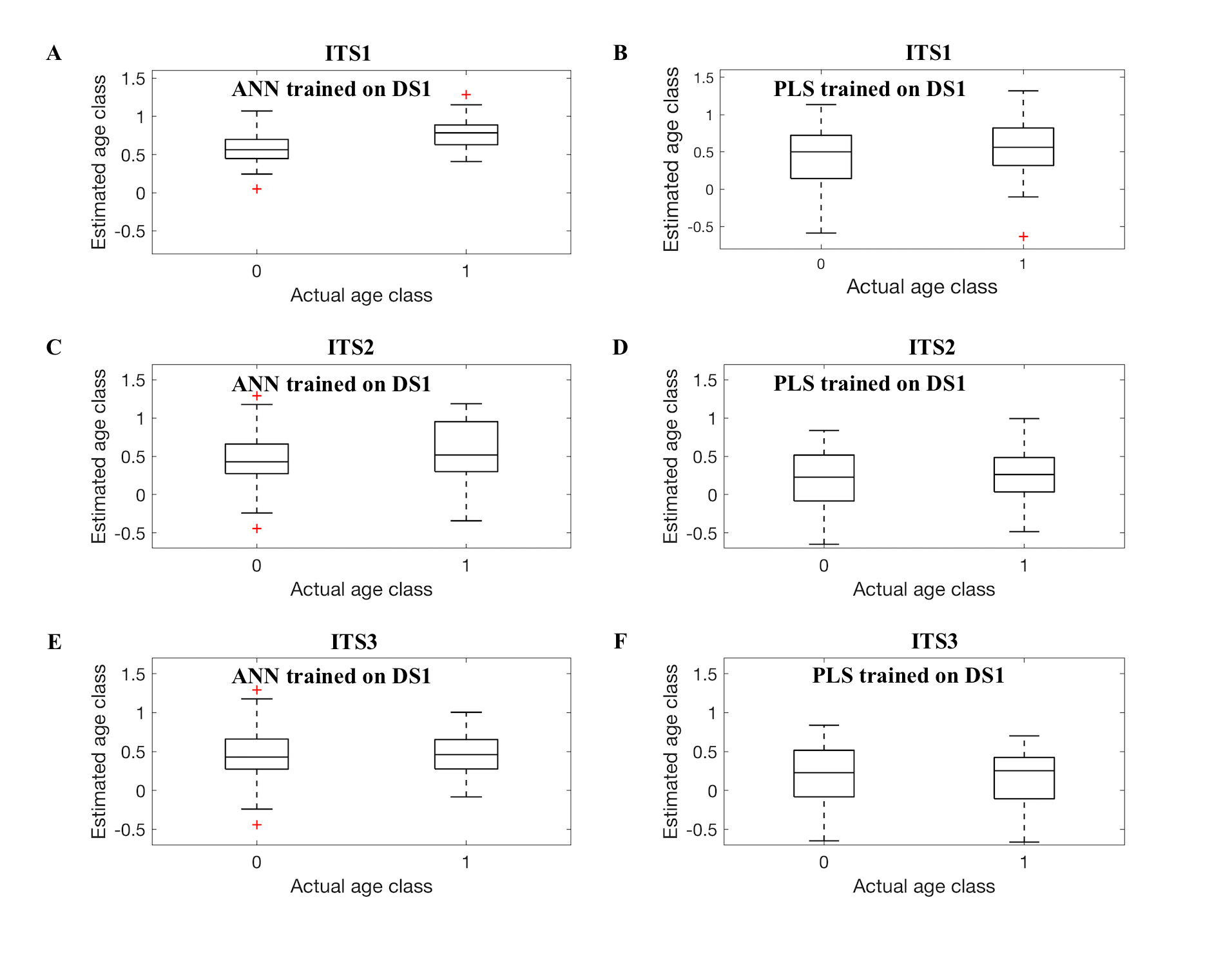

Supplement: S11 Appendix — (ZIP) [file pone.0209451.s027.zip › S11_Appendix/S26_Fig_DS1_ON_ITS1_ITS3_Binary.tif]

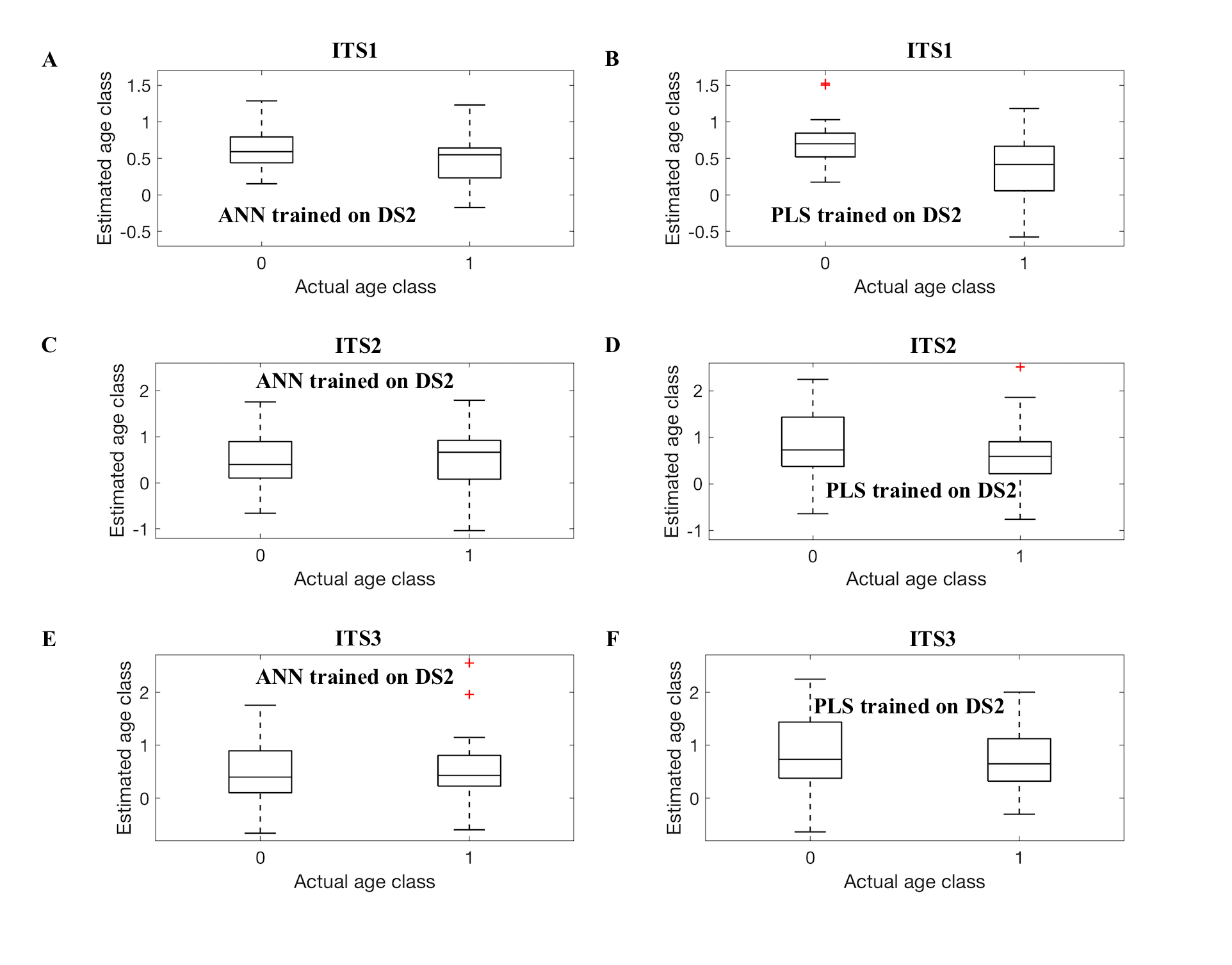

Supplement: S11 Appendix — (ZIP) [file pone.0209451.s027.zip › S11_Appendix/S27_Fig_DS2_ON_ITS1_ITS3_Binary.tif]

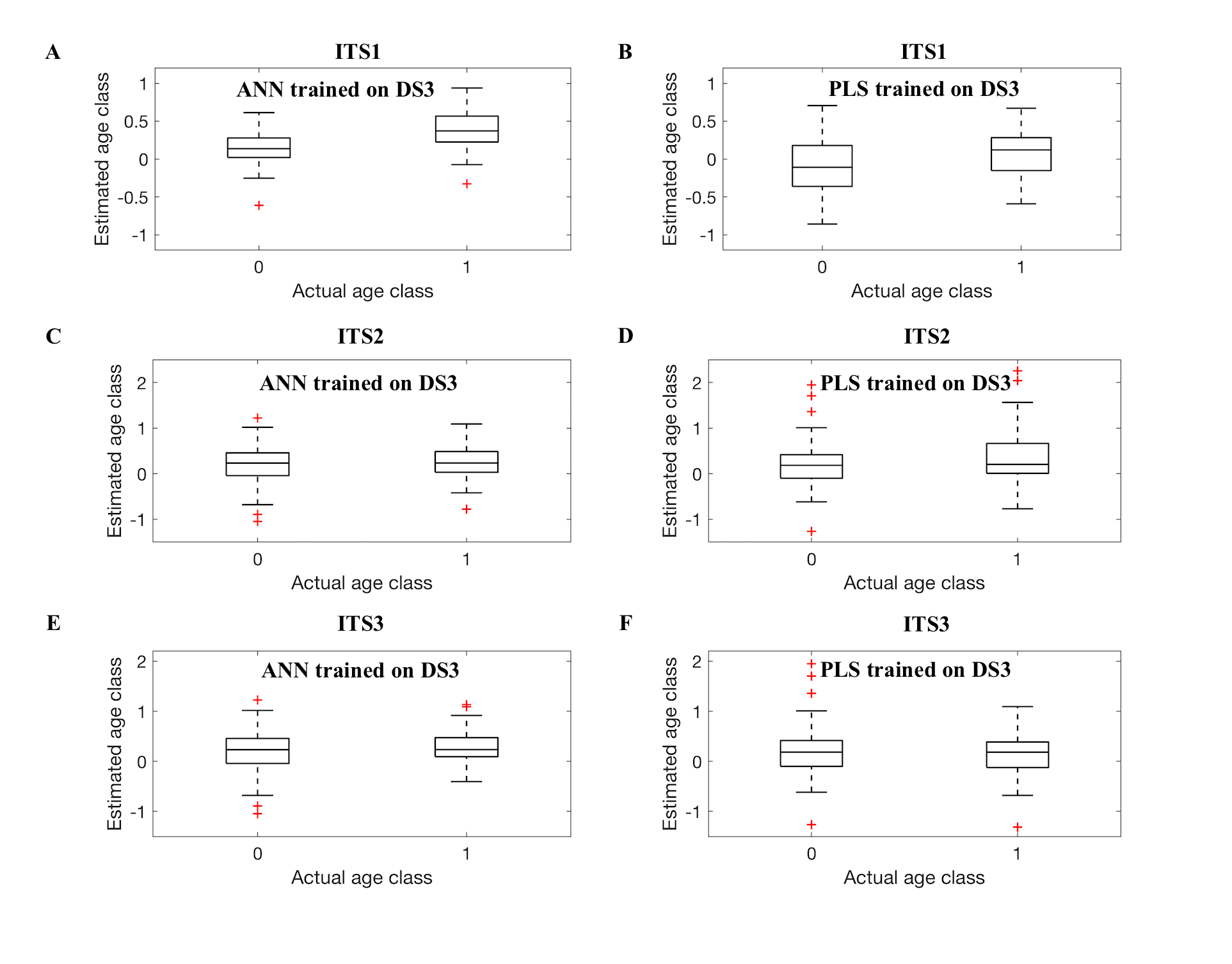

Supplement: S11 Appendix — (ZIP) [file pone.0209451.s027.zip › S11_Appendix/S28_Fig_DS3_ON_ITS1_ITS3_Binary.tif]

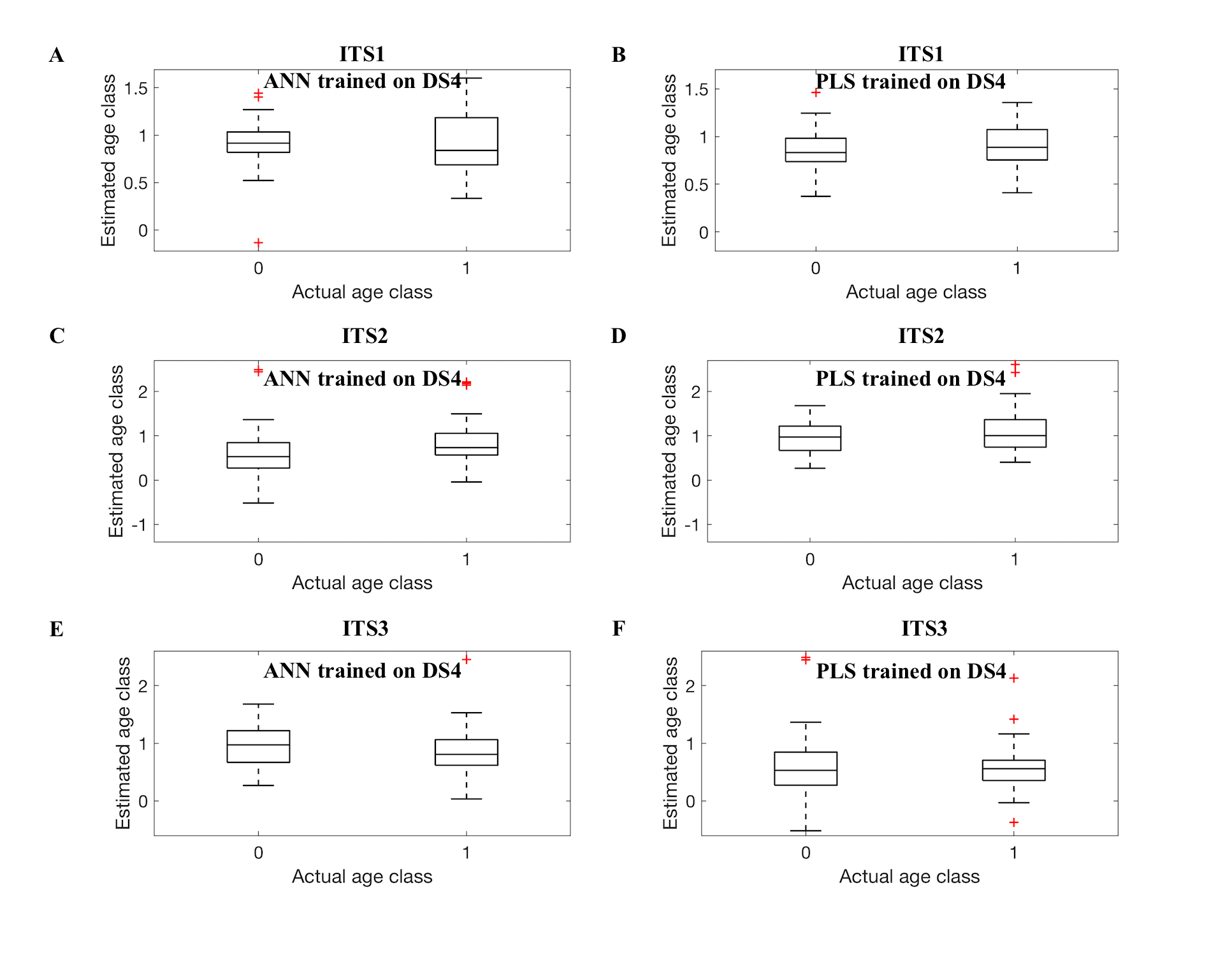

Supplement: S11 Appendix — (ZIP) [file pone.0209451.s027.zip › S11_Appendix/S29_Fig_DS4_ON_ITS1_ITS3_Binary.tif]

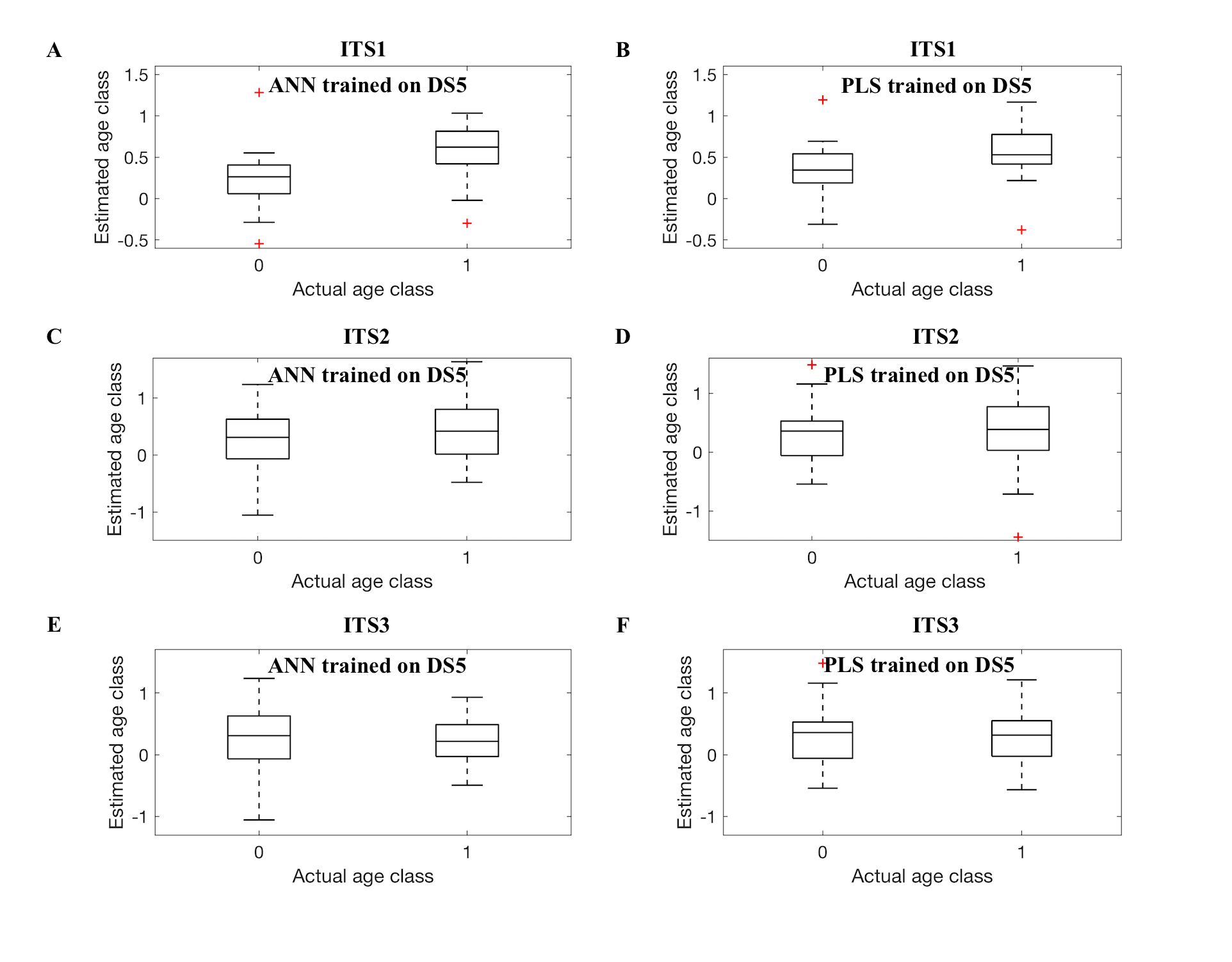

Supplement: S11 Appendix — (ZIP) [file pone.0209451.s027.zip › S11_Appendix/S30_Fig_DS5_ON_ITS1_ITS3_Binary.tif]

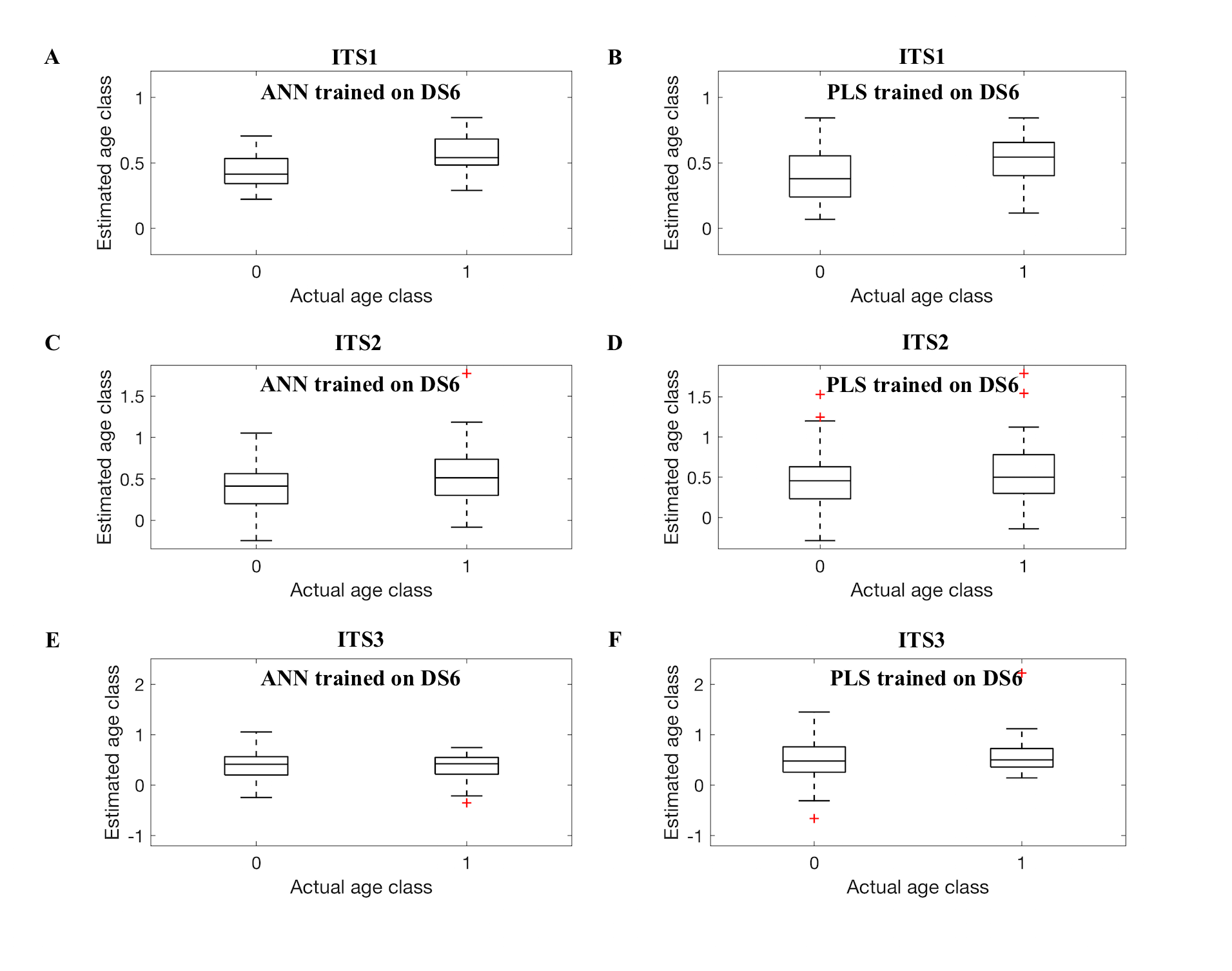

Supplement: S11 Appendix — (ZIP) [file pone.0209451.s027.zip › S11_Appendix/S31_Fig_DS6_ON_ITS1_ITS3_Binary.tif]

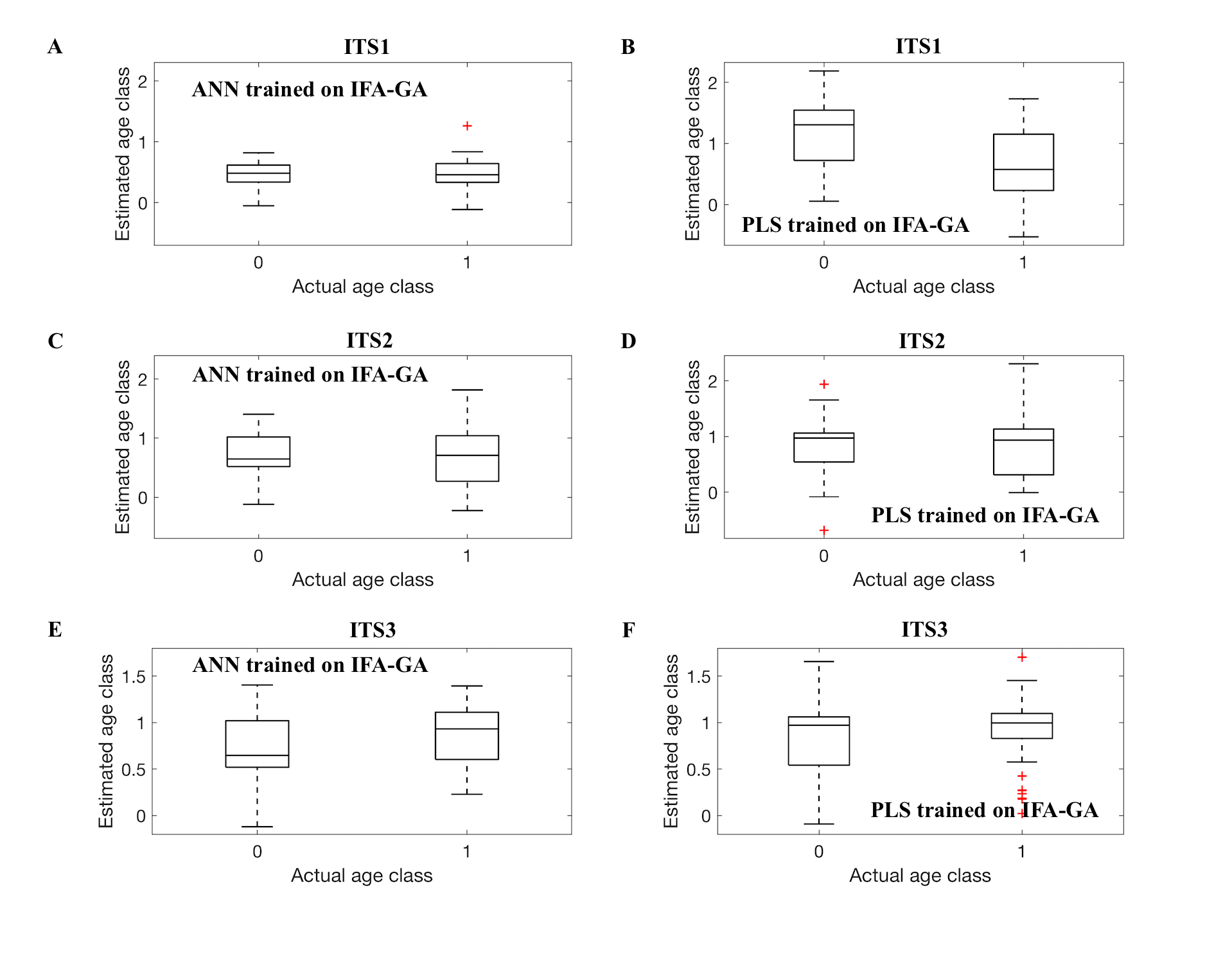

Supplement: S11 Appendix — (ZIP) [file pone.0209451.s027.zip › S11_Appendix/S32_Fig_IFA-GA_ON_ITS1_ITS3_Binary.tif]
