# Supplementary material for: Age grading An. gambiae and An. arabiensis using near infrared spectra and artificial neural networks
Source: PLoS One. 2019 Aug 14;14(8):e0209451. doi: 10.1371/journal.pone.0209451 (PMC6693756; doi:10.1371/journal.pone.0209451)
Supplement: S1 Table — Numbers in brackets are references of the studies where dataset is originally published. (DOCX) [file pone.0209451.s008.docx]

**S1 Table: List and summary of mosquito datasets used to test reproducibility of our study. Numbers in brackets are references of the studies where dataset is originally published.**

| Species | Dataset ID | Description | |
| --- | --- | --- | --- |
| *An.gambiae*  (33)  DS4 | DS1 | 1^st^ generation mosquitoes emerged from wild larvae collected in 2013 at Soumousso (DS1) and in 2014 at Kodeni (DS2) in southwestern Burkina Faso and reared under ambient conditions. | Spectra collection at 3, 6, 9, 12, and 15 days using LabSpec4i spectrometer (ASD Inc., Boulder, CO, USA).  Killed using either triethylamine, Chloroform (DS1, DS2, DS3) and by freezing at -20 C for ~30 minutes and left to equilibrate for another 30 min in a room temperature before spectra collection (DS4). |
|  | DS2 |  |  |
|  | DS3 | Mosquitoes from a colony established in 2015 with original larvae collected in Bukinafaso (DS3) and from a colony established in 1975 (DS4). Both colonies reared at Colorado state university (CSU) at 28 ± 2 °C and 80% humidity under a 14:10 light: dark photoperiod |  |
|  | DS4 |  |  |
|  | DS5 | DS1 and DS2 combined |  |
|  | DS6 | DS1, DS2, and DS3 combined |  |
| *An. arabiensis*  (20, 32) | DS7 (32) | Reared at Ifakara Health Institute in semi-field systems under ambient conditions.  Spectra collection at 3, 5, 8 and 11 days old using QualitySpec Pro Spectrometer (ASD Inc, Boulder, CO).  Killed using chloroform before spectra collection | |
|  | DS8 (20) | Wild larvae and pupae collected in Pemba from different mosquito breeding sites and reared under ambient conditions.  Spectra collection at 1, 3, 5, 7, 9 and 14 days old using LabSpec 5000 NIR spectrometer (ASD Inc, Boulder, CO).  RNAlater to preserve samples before spectra collection.  Pyrethoid resistant. | |
| *Aedes aegypti*  (24) | DS9 | Wolbachia free males | Reared at the insectary of QIMR Berghofer Medical Research Institute, Australia, in separate rooms under identical conditions; 27°C, 70% humidity, 12:12 hr day:night lighting.  Spectra collection at 1, 5, 9, 10, 15, 19, 20 and 25 days old using LabSpec 5000 NIR spectrometer model (ASD Inc, Boulder, CO)  Preserved in RNAlater before spectra collection |
|  | DS10 | Wolbachia free females |  |
|  | DS11 | wMelPop infected males |  |
|  | DS12 | wMelPop infected female |  |
|  | DS13 | wMel infected males |  |
|  | DS14 | wMel infected female |  |
|  | DS15 | DS9, DS11 and DS13 combined |  |
|  | DS16 | DS10, DS12 and DS14 combined |  |
| *Ae albopictus* (34) | DS17 | Reared at the insectary in QIMR Berghofer Medical Research Institute, Australia at 27 °C, 70% humidity with 12:12 hr day:night lighting and 30 min dawn/dusk periods.  Spectra collection at 3, 7, 9, 13, 16, 20 and 25 days old using LabSpec 4S*i* NIR spectrometer (ASD Inc, Boulder, CO).  Preserved in RNAlater before spectra collection | |
