# Supplementary material for: Age grading An. gambiae and An. arabiensis using near infrared spectra and artificial neural networks
Source: PLoS One. 2019 Aug 14;14(8):e0209451. doi: 10.1371/journal.pone.0209451 (PMC6693756; doi:10.1371/journal.pone.0209451)
Supplement: S2 Table — (DOCX) [file pone.0209451.s009.docx]

**S2 Table: Number of mosquito in datasets used to test reproducibility of our study.**

| Dataset | 1d | 3d | 5d | 6d | 7d | 8d | 9d | 10d | 11d | 12d | 13d | 14d | 15d | 16d | 17d | 19d | 20d | 21d | 25d |
| --- | --- | --- | --- | --- | --- | --- | --- | --- | --- | --- | --- | --- | --- | --- | --- | --- | --- | --- | --- |
|  |  |  |  |  |  |  |  |  |  |  |  |  |  |  |  |  |  |  |  |
| DS1 | - | 48 | - | 50 | - | - | 48 | - | - | 49 | - | - | 28 | - | - | - | - | - | - |
| DS2 | - | 39 | - | 40 | - | - | 37 | - | - | 40 | - | - | 38 | - | - | - | - | - | - |
| DS3 | - | 50 | - | 49 | - | - | 15 | - | - | 45 | - | - | 42 | - | - | - | - | - | - |
| DS4 | - | 50 | - | 50 | - | - | 50 | - | - | 50 | - | - | 50 | - | - | - | - | - | - |
| DS5 | - | 87 | - | 90 | - | - | 85 | - | - | 89 | - | - | 66 | - | - | - | - | - | - |
| DS6 | - | 137 | - | 139 | - | - | 100 | - | - | 106 | - | - | 108 | - | - | - | - | - | - |
| DS7 | - | 104 | 122 | - | - | 92 | - | - | 209 | - | - | - | - | - | - | - | - | - | - |
| DS8 | 9 | 61 | 51 | - | 101 | - | 47 | - | - | - | 1 | 9 | - | - | - | - | - | - | - |
| DS9 | 25 | - | 30 | - | - | - | 76 | - | - | - | 66 | - | - | - | 95 | - | - | 55 | 36 |
| DS10 | 72 | - | 110 | - | - | - | 71 | - | - | - | 38 | - | - | - | 114 | - | - | 30 | 73 |
| DS11 | 43 | - | 48 | - | - | - | - | 45 | - | - | - | - | 46 | - | - | 51 | - | - | - |
| DS12 | 40 | - | 45 | - | - | - | - | 46 | - | - | - | - | 45 | - | - | 53 | - | - | - |
| DS13 | 48 | - | 60 | - | - | - | - | 43 | - | - | - | - | 50 | - | - | 29 | - | - | - |
| DS14 | 52 | - | 45 | - | - | - | - | 45 | - | - | - | - | 49 | - | - | 44 | 49 | - | - |
| DS15 | 116 | - | 138 | - | - | - | 76 | 88 | - | - | 66 | - | 96 | - | 95 | 80 | 47 | 55 | 36 |
| DS16 | 164 | - | 200 | - | - | - | 71 | 91 | - | - | 38 |  | 94 | - | 114 | 97 | 49 | 30 | 73 |
| DS17 | 49 | 51 | 51 | - | 53 | - | 100 | - |  |  | 102 | - | - | 48 | - | - | 54 | - | 66 |
