# Supplementary material for: Age grading An. gambiae and An. arabiensis using near infrared spectra and artificial neural networks
Source: PLoS One. 2019 Aug 14;14(8):e0209451. doi: 10.1371/journal.pone.0209451 (PMC6693756; doi:10.1371/journal.pone.0209451)
Supplement: S3 Table — (DOCX) [file pone.0209451.s010.docx]

**S3 Table: Percentage of mosquitoes in each age group correctly classified when ANN and PLS regression models were interpreted as binary classifiers.**

Actual age (in days)

| Model prediction  ($<7,\geq7d$) | 1 | 3 | 5 | 7 | 9 | 11 | 15 | 20 | 25 |  |
| --- | --- | --- | --- | --- | --- | --- | --- | --- | --- | --- |
| An.gambiae | ANN | **100%**  $(\frac{29}{29} )$ | **100%**  $(\frac{45}{45})$ | **94.3%**  $(\frac{33}{35} )$ | **48.8%**  $(\frac{20}{41} )$ | **97.1%**  $(\frac{34}{35} )$ | **100%**  $(\frac{29}{29} )$ | **100%**  $(\frac{36}{36} )$ | **100%**  $(\frac{28}{28} )$ |  |
|  | PLS | **86%**  $(\frac{25}{29} )$ | **82.2%**  $(\frac{37}{45} )$ | **40%**  $(\frac{14}{35} )$ | **82.9%**  $(\frac{34}{41} )$ | **88%**  $(\frac{31}{35} )$ | **93.1%**  $(\frac{27}{29} )$ | **100%**  $(\frac{36}{36} )$ | **100%**  $(\frac{28}{28} )$ |  |
|  |  |  |  |  |  |  |  |  |  |  |
| An. arabiensis | ANN | **100%**  $(\frac{43}{43} )$ | **90%**  $(\frac{36}{40} )$ | **61%**  $(\frac{24}{39} )$ | **72.3%**  $(\frac{34}{47} )$ | **97.1%**  $(\frac{34}{35} )$ | **100%**  $(\frac{45}{45} )$ | **100%**  $(\frac{37}{37} )$ | **100%**  $(\frac{38}{38} )$ | **100%**  $(\frac{38}{38} )$ |
|  | PLS | **93%**  $(\frac{40}{43} )$ | **57.5%**  $(\frac{23}{40} )$ | **25.6%**  $(\frac{10}{39} )$ | **68%**  $(\frac{32}{47} )$ | **71.4%**  $(\frac{25}{35} )$ | **100%**  $(\frac{45}{45} )$ | **100%**  $(\frac{37}{37} )$ | **100%**  $(\frac{38}{38} )$ | **100%**  $(\frac{38}{38} )$ |
