# Supplementary material for: Age grading An. gambiae and An. arabiensis using near infrared spectra and artificial neural networks
Source: PLoS One. 2019 Aug 14;14(8):e0209451. doi: 10.1371/journal.pone.0209451 (PMC6693756; doi:10.1371/journal.pone.0209451)
Supplement: S4 Table — Results from ten-fold Monte Carlo cross-validation. (DOCX) [file pone.0209451.s011.docx]

S4 **Table: Reproducibility analysis of PLS and ANN regression models on estimating age of *An. gambiae* and *An. arabiensis* in different datasets already used in other publications. Results from ten-fold Monte Carlo cross-validation.**

| Species | Model estimation | Metric | Model architecture | | P-value  (two tail) | P-value  (one tail) |
| --- | --- | --- | --- | --- | --- | --- |
|  |  |  | PLS | ANN |  |  |
| DS1    (N = 223) | Actual age | RMSE | 3.07 $\pm$ 0.3 | 2.6 $\pm$ 0.1 | 0.03 | 0.02 |
|  | Age class | Accuracy (%) | 73.1$\pm$ 6.3 | 85.1 $\pm$ 1.7 | 0.012 | 0.01 |
|  |  | Sensitivity (%) | 80.6 $\pm$ 4.2 | 84.2 $\pm$ 2.8 | 0.05 | 0.03 |
|  |  | Specificity (%) | 61.3 $\pm$ 9.4 | 87.5 $\pm$ 2.2 | 0.004 | 0.002 |
|  |  |  |  |  |  |  |
| DS2  (N = 194) | Actual age | RMSE | 2.3 $\pm$ 0.1 | 1.6 $\pm$ 0.2 | 0.008 | < 0.001 |
|  | Age class | Accuracy (%) | 84.7$\pm$ 3.6 | 90.2 $\pm$ 1.9 | 0.002 | 0.001 |
|  |  | Sensitivity (%) | 86.7$\pm$ 5.2 | 93.3 $\pm$ 1.7 | 0.002 | < 0.001 |
|  |  | Specificity (%) | 81.7 $\pm$ 8.1 | 90.2 $\pm$ 2.5 | 0.007 | 0.004 |
| DS3  (N = 201) | Actual age | RMSE | 2.4 $\pm$ 0.1 | 2.3$\pm$ 0.2 | 0.09 | 0.05 |
|  | Age class | Accuracy (%) | 91.5 $\pm$ 4.5 | 93.4 $\pm$ 2.4 | 0.04 | 0.02 |
|  |  | Sensitivity (%) | 94.9$\pm$ 3.4 | 93.9$\pm$ 1.2 | 0.05 | 0.09 |
|  |  | Specificity (%) | 88.4$\pm$ 5.3 | 90.2$\pm$ 2.2 | 0.035 | 0.02 |
|  |  |  |  |  |  |  |
| DS4  (N = 250) | Actual age | RMSE | 2.5 $\pm$ 0.1 | 1.8 $\pm$ 0.1 | 0.017 | 0.009 |
|  | Age class | Accuracy (%) | 84.5 $\pm$ 3.8 | 92.0$\pm$ 2.8 | 0.004 | 0.002 |
|  |  | Sensitivity (%) | 89.6 $\pm$ 3.1 | 93.4$\pm$ 1.4 | 0.043 | 0.022 |
|  |  | Specificity (%) | 77.6 $\pm$ 9.1 | 90.3$\pm$ 2.1 | < 0.001 | < 0.001 |
|  |  |  |  |  |  |  |
| DS5  (N = 417) | Actual age | RMSE | 3.2 $\pm$ 0.1 | 2.5 $\pm$ 0.2 | 0.04 | 0.02 |
|  | Age class | Accuracy (%) | 68.4 $\pm$ 2.8 | 81.7 $\pm$ 2.3 | < 0.001 | 0 |
|  |  | Sensitivity (%) | 80.7 $\pm$ 2.6 | 85.6 $\pm$ 2.4 | 0.028 | 0.014 |
|  |  | Specificity (%) | 51.1 $\pm$ 6.8 | 75.5$\pm$ 2.6 | <0.001 | <0.001 |
|  |  |  |  |  |  |  |
| DS6  (N = 618) | Actual age | RMSE | 3.4 $\pm$ 0.2 | 2.7$\pm$ 0.3 | 0.045 | 0.022 |
|  | Age class | Accuracy (%) | 69.8 $\pm$ 1.2 | 80.1 $\pm$ 2.1 | 0.002 | 0.001 |
|  |  | Sensitivity (%) | 81.3 $\pm$ 2.7 | 87.9 $\pm$ 3.0 | 0.032 | 0.017 |
|  |  | Specificity (%) | 56.7 $\pm$ 2.6 | 75.8 $\pm$ 3.4 | <0.001 | <0.001 |
|  |  |  |  |  |  |  |
| DS7  (N = 527) | Actual age | RMSE | 2.3 $\pm$ 0.3 | 1.8 $\pm$ 0.2 | 0.046 | 0.023 |
|  | Age class | Accuracy (%) | 83.1 $\pm$ 4.1 | 90.2$\pm$ 3.4 | 0.035 | 0.017 |
|  |  | Sensitivity (%) | 80.3 $\pm$ 2.5 | 92.3 $\pm$ 1.7 | <0.001 | <0.001 |
|  |  | Specificity (%) | 86.9 $\pm$ 3.8 | 87.9$\pm$ 3.7 | 0.06 | 0.03 |
|  |  |  |  |  |  |  |
| DS8  (N = 279) | Actual age | RMSE | 2.5 $\pm$ 0.2 | 1.8$\pm$ 0.3 | 0.019 | 0.009 |
|  | Age class | Accuracy (%) | 76.7 $\pm$ 6.2 | 80.6 $\pm$ 2.5 | 0.021 | 0.011 |
|  |  | Sensitivity (%) | 60.8$\pm$ 8.3 | 71.4 $\pm$ 2.7 | <0.001 | <0.001 |
|  |  | Specificity (%) | 82.3$\pm$ 5.8 | 88.2 $\pm$ 3.2 | 0.024 | 0.013 |
