# Supplementary material for: Age grading An. gambiae and An. arabiensis using near infrared spectra and artificial neural networks
Source: PLoS One. 2019 Aug 14;14(8):e0209451. doi: 10.1371/journal.pone.0209451 (PMC6693756; doi:10.1371/journal.pone.0209451)
Supplement: S5 Table — Results from ten-fold Monte Carlo cross-validation. (DOCX) [file pone.0209451.s012.docx]

**S5 Table: Performance analysis of PLS and ANN regression models on estimating age of *Aedes* *albopictus*, *Wolbachia* free and *Wolbachia* infected male and female *Aedes aegypti*. Results from ten-fold Monte Carlo cross-validation.**

| Species | | Model estimation | Metric | Model architecture | | P-value  (two tail) | P-value  (one tail) |
| --- | --- | --- | --- | --- | --- | --- | --- |
|  |  |  |  | PLS | ANN |  |  |
| DS9  (N = 395) | | Actual age | RMSE | 3.8 $\pm$ 0.2 | 2.7 $\pm$ 0.1 | < 0.001 | < 0.001 |
|  |  | Age class | Accuracy (%) | 79.4 $\pm$ 4.6 | 91.9 $\pm$ 1.9 | < 0.001 | < 0.001 |
|  |  |  | Sensitivity (%) | 81.7$\pm$ 3.3 | 95.2 $\pm$ 2.7 | < 0.001 | < 0.001 |
|  |  |  | Specificity (%) | 75.9 $\pm$ 6.2 | 92.0$\pm$ 4.8 | < 0.001 | < 0.001 |
|  | |  |  |  |  |  |  |
| DS10  (N = 600) | | Actual age | RMSE | 5.7 $\pm$ 0.1 | 3.2$\pm$ 0.3 | < 0.001 | < 0.001 |
|  |  | Age class | Accuracy (%) | 77.2$\pm$ 2.0 | 91.7 $\pm$ 2.1 | < 0.001 | < 0.001 |
|  |  |  | Sensitivity (%) | 78.7 $\pm$ 2.2 | 92.6 $\pm$ 1.7 | < 0.001 | < 0.001 |
|  |  |  | Specificity (%) | 72.5$\pm$ 7.4 | 90.0 $\pm$ 3.2 | < 0.001 | < 0.001 |
|  | |  |  |  |  |  |  |
| DS11  (N = 233) | | Actual age | RMSE | 4.7$\pm$ 0.2 | 2.8 $\pm$ 0.1 | < 0.001 | < 0.001 |
|  |  | Age class | Accuracy (%) | 80.3$\pm$ 3.1 | 90.4$\pm$4.1 | < 0.001 | < 0.001 |
|  |  |  | Sensitivity (%) | 87.8$\pm$ 1.2 | 94.1$\pm$ 4.3 | 0.008 | 0.003 |
|  |  |  | Specificity (%) | 78.8$\pm$ 6.3 | 90.9$\pm$ 4.7 | < 0.001 | < 0.001 |
|  | |  |  |  |  |  |  |
| DS12  (N = 229) | | Actual age | RMSE | 4.0$\pm$ 0.2 | 3.1$\pm$ 0.2 | 0.002 | 0.005 |
|  |  | Age class | Accuracy (%) | 78.3$\pm$ 4.1 | 84.3$\pm$ 4.2 | 0.03 | 0.02 |
|  |  |  | Sensitivity (%) | 82.6$\pm$ 3.6 | 88.6$\pm$ 3.2 | 0.012 | 0.023 |
|  |  |  | Specificity (%) | 69.1$\pm$ 8.6 | 83.1$\pm$ 5.3 | < 0.001 | 0.001 |
|  | |  |  |  |  |  |  |
| DS13  (N = 277) | | Actual age | RMSE | 3.7 $\pm$ 0.3 | 2.4 $\pm$ 0.2 | < 0.001 | < 0.001 |
|  |  | Age class | Accuracy (%) | 87.9 $\pm$ 4.3 | 90.5 $\pm$ 2.3 | 0.023 | 0.042 |
|  |  |  | Sensitivity (%) | 89.0 $\pm$ 7.2 | 91.1$\pm$ 2.8 | 0.04 | 0.07 |
|  |  |  | Specificity (%) | 85.2$\pm$ 6.3 | 96.5 $\pm$ 4.3 | < 0.001 | < 0.001 |
|  | |  |  |  |  |  |  |
| DS14  (N = 284) | | Actual age | RMSE | 4.8$\pm$ 0.1 | 3.7$\pm$ 0.2 | 0.002 | 0.001 |
|  |  | Age class | Accuracy (%) | 83.7$\pm$ 3.4 | 87.2 $\pm$ 2.6 | 0.02 | 0.008 |
|  |  |  | Sensitivity (%) | 96.1$\pm$ 2.6 | 88.9 $\pm$ 1.2 | 0.038 | 0.9 |
|  |  |  | Specificity (%) | 62.6$\pm$ 9.7 | 85.2$\pm$ 4.3 | < 0.001 | < 0.001 |
|  | |  |  |  |  |  |  |
| DS15  (N = 905) | Actual age | RMSE | 5.0 $\pm$ 0.1 | 3.6 $\pm$ 0.6 | 0.029 | 0.031 |  |
|  | Age class | Accuracy (%) | 72.9 $\pm$ 1.5 | 82.1$\pm$ 3.6 | < 0.001 | < 0.001 |  |
|  |  | Sensitivity (%) | 73.2 $\pm$ 2.3 | 81.3$\pm$ 3.3 | 0.002 | 0.005 |  |
|  |  | Specificity (%) | 68.9 $\pm$ 2.4 | 83.8$\pm$ 4.9 | < 0.001 | < 0.001 |  |
|  |  |  |  |  |  |  |  |
| DS16  (N = 1113) | Actual age | RMSE | 4.2$\pm$ 0.2 | 2.4 $\pm$ 0.5 | < 0.001 | < 0.001 |  |
|  | Age class | Accuracy (%) | 76.6$\pm$ 2.8 | 84.1$\pm$ 3.1 | < 0.001 | < 0.001 |  |
|  |  | Sensitivity (%) | 78.8$\pm$ 4.3 | 86.4 $\pm$ 2.1 | 0.004 | 0.007 |  |
|  |  | Specificity (%) | 73.3$\pm$ 1.9 | 82.1$\pm$ 6.4 | < 0.001 | < 0.001 |  |
|  |  |  |  |  |  |  |  |
| DS17  (N = 585) | Actual age | RMSE | 4.1$\pm$ 0.3 | 2.3 $\pm$ 0.2 | < 0.001 | < 0.001 |  |
|  | Age class | Accuracy (%) | 87.6 $\pm$ 2.9 | 92.9 $\pm$ 2.1 | 0.006 | 0.012 |  |
|  |  | Sensitivity (%) | 89.9 $\pm$ 3.0 | 92.7 $\pm$ 1.6 | 0.05 | 0.03 |  |
|  |  | Specificity (%) | 85.1$\pm$ 4.3 | 94.3 $\pm$ 2.7 | < 0.001 | < 0.001 |  |
