# Supplementary material for: Age grading An. gambiae and An. arabiensis using near infrared spectra and artificial neural networks
Source: PLoS One. 2019 Aug 14;14(8):e0209451. doi: 10.1371/journal.pone.0209451 (PMC6693756; doi:10.1371/journal.pone.0209451)
Supplement: S6 Table — Results from ten-fold Monte Carlo cross-validation. (DOCX) [file pone.0209451.s013.docx]

**S6 Table: Comparison of accuracies between directly trained binary classifiers and regressers interpreted as binary classifiers. Results from ten-fold Monte Carlo cross-validation**

| Species | Model architecture | Metric | Model type | | P-value  (two tail) | P-value  (one tail) |
| --- | --- | --- | --- | --- | --- | --- |
|  |  |  | DTBC* | RIBC** |  |  |
| *An. gambiae* | PLS | Accuracy (%) | 93.6 $\pm$ 1.2 | 83.9 $\pm$ 2.3 | 3.5 x 10^-05^ | 1.8 x 10^-05^ |
|  |  | Sensitivity (%) | 94.4 $\pm$ 1.6 | 89.0 $\pm$ 2.1 | 1.1 x 10^-03^ | 5.3 x 10^-04^ |
|  |  | Specificity (%) | 92.4 $\pm$ 1.9 | 75.8 $\pm$ 5.2 | 1.3 x 10^-04^ | 6.8 x 10^-05^ |
|  | ANN | Accuracy (%) | 99.4 $\pm$ 1.0 | 93.7 $\pm$ 1.0 | 2.3 x 10^-19^ | 1.2 x 10^-19^ |
|  |  | Sensitivity (%) | 99.3 $\pm$ 1.4 | 92.5 $\pm$ 1.6 | 7.3 x 10^-07^ | 3.7 x 10^-07^ |
|  |  | Specificity (%) | 99.5 $\pm$ 0.7 | 95.6 $\pm$ 1.8 | 2.2 x 10^-03^ | 1.1 x 10^-03^ |
| *An. arabiensis* | PLS | Accuracy (%) | 88.7 $\pm$ 1.1 | 80.3 $\pm$ 2.1 | 6.9 x 10^-08^ | 3.4 x 10^-08^ |
|  |  | Sensitivity (%) | 95.4 $\pm$ 1.4 | 90.5 $\pm$ 1.9 | 2.3 x 10^-04^ | 1.2 x 10^-04^ |
|  |  | Specificity (%) | 75.2$\pm$ 3.4 | 60.3 $\pm$ 4.2 | 5.5 x 10^-05^ | 2.8 x 10^-05^ |
|  | ANN | Accuracy (%) | 99.0 $\pm$ 0.6 | 90.2 $\pm$ 1.7 | 1.8 x 10^-21^ | 8.8 x 10^-22^ |
|  |  | Sensitivity (%) | 99.5 $\pm$ 0.5 | 91.7 $\pm$ 3.3 | 3.2 x 10^-05^ | 1.6 x 10^-05^ |
|  |  | Specificity (%) | 98.3$\pm$ 1.3 | 88.4 $\pm$ 3.9 | 1.1 x 10^-04^ | 5.3 x 10^-05^ |

*Directly trained binary classifier

**Regresser interpreted as binary classifier
