# Supplementary material for: Age grading An. gambiae and An. arabiensis using near infrared spectra and artificial neural networks
Source: PLoS One. 2019 Aug 14;14(8):e0209451. doi: 10.1371/journal.pone.0209451 (PMC6693756; doi:10.1371/journal.pone.0209451)
Supplement: S7 Table — (DOCX) [file pone.0209451.s014.docx]

**S7 Table: Comparison of the accuracy of ANN and PLS classification models on *An. gambiae* and *An. arabiensis* in datasets from other published studies.**

| Dataset | Metric | Model architecture | | P-value  (two-tail) | P-value  (one-tail) |
| --- | --- | --- | --- | --- | --- |
|  |  | PLS | ANN |  |  |
| DS1  (N = 223) | Accuracy (%) | 78.8$\pm$ 3.4 | 88.9 $\pm$ 2.1 | < 0.001 | < 0.001 |
|  | Sensitivity (%) | 80.9 $\pm$ 4.6 | 90.7 $\pm$ 1.6 | < 0.001 | < 0.001 |
|  | Specificity (%) | 80.7$\pm$ 2.4 | 86.6 $\pm$ 1.4 | < 0.001 | < 0.001 |
|  |  |  |  |  |  |
| DS2  (N = 194) | Accuracy (%) | 87.8 $\pm$3.6 | 92.9$\pm$ 2.7 | <0.001 | <0.001 |
|  | Sensitivity (%) | 89.3 $\pm$ 5.3 | 92.7 $\pm$ 2.6 | 0.046 | 0.023 |
|  | Specificity (%) | 86.2$\pm$ 5.8 | 92.9$\pm$ 2.9 | <0.001 | <0.001 |
|  |  |  |  |  |  |
| DS3  N = 201 | Accuracy (%) | 93.4 $\pm$ 5.1 | 96.7 $\pm$ 1.2 | <0.001 | <0.001 |
|  | Sensitivity (%) | 91.3 $\pm$ 7.6 | 93.9 $\pm$ 1.4 | <0.001 | <0.001 |
|  | Specificity (%) | 95.6$\pm$ 2.9 | 98.7$\pm$ 2.3 | 0.042 | 0.022 |
|  |  |  |  |  |  |
| DS4  N = 250 | Accuracy (%) | 88.0 $\pm$ 3.1 | 94.2 $\pm$ 2.0 | <0.001 | <0.001 |
|  | Sensitivity (%) | 86.0 $\pm$ 6.2 | 96.7 $\pm$ 1.2 | < 0.001 | <0.001 |
|  | Specificity (%) | 90.1$\pm$ 7.1 | 94.7 $\pm$ 1.8 | 0.005 | 0.003 |
|  |  |  |  |  |  |
| DS5  N = 417 | Accuracy (%) | 73.8 $\pm$ 5.6 | 78.6 $\pm$ 2.4 | <0.001 | <0.001 |
|  | Sensitivity (%) | 82.7 $\pm$ 2.8 | 87.1 $\pm$ 1.8 | 0.032 | 0.017 |
|  | Specificity (%) | 67.3$\pm$ 7.1 | 72.9$\pm$ 4.8 | 0.034 | 0.018 |
|  |  |  |  |  |  |
| DS6  N = 417 | Accuracy (%) | 75.3 $\pm$ 2.8 | 83.3 $\pm$ 3.0 | <0.001 | <0.001 |
|  | Sensitivity (%) | 78.9 $\pm$ 4.3 | 84.5 $\pm$ 2.6 | 0.003 | 0.002 |
|  | Specificity (%) | 70.9$\pm$ 2.2 | 80.7 $\pm$ 2.3 | <0.001 | <0.001 |
|  |  |  |  |  |  |
| DS7  (N = 527) | Accuracy (%) | 87$.4 \pm$ 1.7 | 91.7 $\pm$ 3.2 | 0.006 | 0.003 |
|  | Sensitivity (%) | 88$.8 \pm$ 2.1 | 95.6 $\pm$ 2.8 | 0.004 | 0.002 |
|  | Specificity (%) | 85.6 $\pm$ 3.4 | 90.1 $\pm$ 1.1 | 0.002 | 0.001 |
|  |  |  |  |  |  |
| DS8  (N = 279) | Accuracy (%) | 83.3 $\pm$ 4.3 | 90.4 $\pm$ 2.3 | < 0.001 | <0.001 |
|  | Sensitivity (%) | 75.7 $\pm$ 4.8 | 87.5 $\pm$ 3.1 | < 0.001 | < 0.001 |
|  | Specificity (%) | 88.5 $\pm$ 3.4 | 94.2 $\pm$ 2.9 | 0.005 | 0.002 |
