# Supplementary material for: Age grading An. gambiae and An. arabiensis using near infrared spectra and artificial neural networks
Source: PLoS One. 2019 Aug 14;14(8):e0209451. doi: 10.1371/journal.pone.0209451 (PMC6693756; doi:10.1371/journal.pone.0209451)
Supplement: S8 Table — (DOCX) [file pone.0209451.s015.docx]

**Table 8: Comparison of the accuracies of directly trained ANN and PLS classification models on *Aedes aegypti* and *Aedes albopictus* in datasets from other published studies.**

| Dataset | Metric | Model architecture | | P-value  (two-tail) | P-value  (one-tail) |
| --- | --- | --- | --- | --- | --- |
|  |  | PLS | ANN |  |  |
| DS9  (N = 395) | Accuracy (%) | 86.9 $\pm$ 2.8 | 92.4 $\pm$ 2.5 | < 0.001 | < 0.001 |
|  | Sensitivity (%) | 93.3 $\pm$ 4.3 | 94.5$\pm$ 2.8 | 0.22 | 0.89 |
|  | Specificity (%) | 82.3$\pm$ 2.2 | 88.9 $\pm$ 1.4 | < 0.001 | < 0.001 |
| DS10  (N = 600) | Accuracy (%) | 91.9 $\pm$ 1.3 | 97.4 $\pm$ 1.2 | <0.001 | <0.001 |
|  | Sensitivity (%) | 93.9 $\pm$ 1.9 | 96.5 $\pm$ 1.0 | 0.034 | 0.05 |
|  | Specificity (%) | 89 2 $\pm$ 1.7 | 94.6 $\pm$ 1.8 | 0.005 | 0.008 |
| DS11  (N = 233) | Accuracy (%) | 85.7 $\pm$ 2.1 | 94.6 $\pm$ 1.3 | < 0.001 | < 0.001 |
|  | Sensitivity (%) | 89.1$\pm$ 3.2 | 96.4$\pm$ 1.9 | 0.04 | 0.06 |
|  | Specificity (%) | 83.2 $\pm$ 5.6 | 92.1$\pm$ 2.8 | < 0.001 | < 0.001 |
| DS12  (N = 229) | Accuracy (%) | 89.9 $\pm$ 6.5 | 95.3 $\pm$ 3.4 | < 0.001 | < 0.001 |
|  | Sensitivity (%) | 89.9 $\pm$ 6.7 | 93.6 $\pm$ 3.1 | 0.04 | 0.06 |
|  | Specificity (%) | 80.0$\pm$ 7.5 | 91.2 $\pm$ 3.6 | < 0.001 | < 0.001 |
| DS13  (N = 277) | Accuracy (%) | 91.2 $\pm$ 2.7 | 95.9 $\pm$ 2.8 | 0.01 | 0.007 |
|  | Sensitivity (%) | 94.6 $\pm$ 2.2 | 94.4$\pm$ 2.9 | 0.72 | 0.36 |
|  | Specificity (%) | 86.$4\pm$ 6.8 | 98.5 $\pm$ 1.7 | < 0.001 | < 0.001 |
| DS14  (N = 284) | Accuracy (%) | 83.7 $\pm$ 3.6 | 92.9 $\pm$ 2.4 | < 0.001 | < 0.001 |
|  | Sensitivity (%) | 91.3 $\pm$ 1.1 | 98.3 $\pm$ 1.2 | < 0.001 | < 0.001 |
|  | Specificity (%) | 67.$8\pm$ 7.2 | 87.8 $\pm$ 2.8 | < 0.001 | < 0.001 |
| DS15  (N = 905) | Accuracy (%) | 84.2 $\pm$ 1.6 | 90.8 $\pm$ 1.2 | 0.008 | 0.005 |
|  | Sensitivity (%) | 89.0 $\pm$ 3.1 | 90.5 $\pm$ 2.6 | 0.277 | 0.862 |
|  | Specificity (%) | 73.$6\pm$ 5.7 | 89.4 $\pm$ 1.1 | < 0.001 | < 0.001 |
| DS16  (N = 1113) | Accuracy (%) | 81.1$\pm$ 1.5 | 92.9 $\pm$ 2.4 | < 0.001 | < 0.001 |
|  | Sensitivity (%) | 88.4 $\pm$ 1.2 | 96.7 $\pm$ 2.1 | 0.003 | 0.002 |
|  | Specificity (%) | 71.7$\pm$ 3.4 | 89.8 $\pm$ 2.7 | < 0.001 | < 0.001 |
| DS17  (N = 585) | Accuracy (%) | 89.8 $\pm$ 2.1 | 95.9 $\pm$ 2.2 | 0.021 | 0.011 |
|  | Sensitivity (%) | 95.6 $\pm$ 1.5 | 98.3 $\pm$ 0.4 | 0.03 | 0.02 |
|  | Specificity (%) | 80.$7\pm$ 6.6 | 94.6 $\pm$ 2.1 | < 0.001 | < 0.001 |
|  |  |  |  |  |  |
