# Supplementary material for: Age grading An. gambiae and An. arabiensis using near infrared spectra and artificial neural networks
Source: PLoS One. 2019 Aug 14;14(8):e0209451. doi: 10.1371/journal.pone.0209451 (PMC6693756; doi:10.1371/journal.pone.0209451)
Supplement: S9 Table — (DOCX) [file pone.0209451.s016.docx]

S9 Table: Results when both regression and directly trained binary classifiers trained on IFA-GA and IFA-ARA datasets were tested on DS1- 4 and DS7-8, respectively, as independent test sets.

| Training set | Test set | Model Type | Metric | Model architecture | |
| --- | --- | --- | --- | --- | --- |
|  |  |  |  | PLS | ANN |
| IFA-GA | DS1 | Regression | RMSE | 5.8 | **4.8** |
|  |  | Classification | Accuracy (%) | 47.9 | **60.7** |
|  |  |  | Sensitivity (%) | 36.8 | **65.6** |
|  |  |  | Specificity (%) | **62.2** | 56.5 |
|  |  |  |  |  |  |
|  | DS2 | Regression | RMSE | 5.4 | **4.9** |
|  |  | Classification | Accuracy (%) | 49.5 | **69.8** |
|  |  |  | Sensitivity (%) | 26.1 | **63.4** |
|  |  |  | Specificity (%) | **83.5** | 77.9 |
|  |  |  |  |  |  |
|  | DS3 | Regression | RMSE | 5.82 | **4.1** |
|  |  | Classification | Accuracy (%) | 63.7 | **77.8** |
|  |  |  | Sensitivity (%) | 66.7 | **76.5** |
|  |  |  | Specificity (%) | 60.6 | **79.4** |
|  |  |  |  |  |  |
|  | DS4 | Regression | RMSE | 5.7 | **4.6** |
|  |  | Classification | Accuracy (%) | 56.9 | **73.7** |
|  |  |  | Sensitivity (%) | 66.4 | **77.3** |
|  |  |  | Specificity (%) | 48.0 | **70.8** |
| IFA-ARA | DS7 | Regression | RMSE | 3.8 | **2.9** |
|  |  | Classification | Accuracy (%) | 71.4 | **82.4** |
|  |  |  | Sensitivity (%) | 67.8 | **80.5** |
|  |  |  | Specificity (%) | 75.3 | **83.2** |
|  |  |  |  |  |  |
|  | DS8 | Regression | RMSE | 5.9 | **4.1** |
|  |  | Classification | Accuracy (%) | 48.7 | **72.6** |
|  |  |  | Sensitivity (%) | 32.3 | **73.5** |
|  |  |  | Specificity (%) | 70.3 | **72.1** |
