# Supplementary material for: Age grading An. gambiae and An. arabiensis using near infrared spectra and artificial neural networks
Source: PLoS One. 2019 Aug 14;14(8):e0209451. doi: 10.1371/journal.pone.0209451 (PMC6693756; doi:10.1371/journal.pone.0209451)
Supplement: S11 Appendix — (ZIP) [file pone.0209451.s027.zip › S11_Appendix/S10_Table.docx]

S10 Table: Results when our models trained on datasets DS1 - DS6 and

IFA-GA were applied on independent test sets (nulliparous vs. sporozoite positive field samples) as presented by Krajacich et al. 2017.

| **Training set** | **Model** | **Accuracy on independent test sets** | | |
| --- | --- | --- | --- | --- |
|  |  | **ITS1** | **ITS2** | **ITS3** |
| **DS1** | ANN | **65.2** | **68.1** | **54.3** |
|  | PLS | 50.7 | 55.3 | 44.8 |
|  |  |  |  |  |
| **DS2** | ANN | **50.7** | **60.1** | **50.0** |
|  | PLS | 40.4 | 46.6 | 47.9 |
|  |  |  |  |  |
| **DS3** | ANN | **60.9** | **58.5** | **51.0** |
|  | PLS | 52.2 | 54.5 | 47.9 |
|  |  |  |  |  |
| **DS4** | ANN | **49.3** | **59.1** | 46.9 |
|  | PLS | 46.4 | 46.5 | **54.2** |
|  |  |  |  |  |
| **DS5** | ANN | **78.6** | 56.8 | **50.8** |
|  | PLS | 62.3 | **57.9** | 49.9 |
|  |  |  |  |  |
| **DS6** | ANN | **69.6** | **60.1** | 50.0 |
|  | PLS | 65.2 | 56.9 | **51.0** |
|  |  |  |  |  |
| **IFA-GA** | ANN | **50.8** | **42.1** | **54.1** |
|  | PLS | 37.7 | 38.6 | 52.2 |
